# Genomic and proteomic characterization of vB_SauM-UFV_DC4, a novel *Staphylococcus* jumbo phage

**DOI:** 10.1007/s00253-023-12743-6

**Published:** 2023-09-23

**Authors:** Jéssica Duarte da Silva, Luís D. R. Melo, Sílvio B. Santos, Andrew M. Kropinski, Mariana Fonseca Xisto, Roberto Sousa Dias, Isabela da Silva Paes, Marcella Silva Vieira, José Júnior Ferreira Soares, Davide Porcellato, Vinícius da Silva Duarte, Sérgio Oliveira de Paula

**Affiliations:** 1 Department of Microbiology, Federal University of Viçosa, Av. Peter Henry Rolfs, S/N, Campus Universitário, Viçosa, Minas Gerais 36570-900 Brazil; 2 Centre of Biological Engineering – CEB, University of Minho, 4710-057 Braga, Portugal; 3 LABBELS – Associate Laboratory, Braga, Portugal; 4 Department of Pathobiology, University of Guelph, Guelph, ON N1G 2W1 Canada; 5 Department of General Biology, Federal University of Viçosa, Av. Peter Henry Rolfs, S/N, Campus Universitário, Viçosa, Minas Gerais 36570-900 Brazil; 6 Faculty of Chemistry, Biotechnology and Food Science, Norwegian University of Life Sciences, P.O. Box 5003, 1432 Ås, Norway

**Keywords:** Jumbo phages, *Staphylococcus aureus*, VAPGH, Endolysin, Bovine mastitis

## Abstract

**Abstract:**

*Staphylococcus* *aureus* is one of the most relevant mastitis pathogens in dairy cattle, and the acquisition of antimicrobial resistance genes presents a significant health issue in both veterinary and human fields. Among the different strategies to tackle *S*. *aureus* infection in livestock, bacteriophages have been thoroughly investigated in the last decades; however, few specimens of the so-called jumbo phages capable of infecting *S*. *aureus* have been described. Herein, we report the biological, genomic, and structural proteomic features of the jumbo phage vB_SauM-UFV_DC4 (DC4). DC4 exhibited a remarkable killing activity against *S*. *aureus* isolated from the veterinary environment and stability at alkaline conditions (pH 4 to 12). The complete genome of DC4 is 263,185 bp (GC content: 25%), encodes 263 predicted CDSs (80% without an assigned function), 1 tRNA (Phe-tRNA), multisubunit RNA polymerase, and an RNA-dependent DNA polymerase. Moreover, comparative analysis revealed that DC4 can be considered a new viral species belonging to a new genus DC4 and showed a similar set of lytic proteins and depolymerase activity with closely related jumbo phages. The characterization of a new *S*. *aureus* jumbo phage increases our understanding of the diversity of this group and provides insights into the biotechnological potential of these viruses.

**Key points:**

• *vB_SauM-UFV_DC4 is a new viral species belonging to a new genus within the class Caudoviricetes.*

• *vB_SauM-UFV_DC4 carries a set of RNA polymerase subunits and an RNA-directed DNA polymerase.*

• *vB_SauM-UFV_DC4 and closely related jumbo phages showed a similar set of lytic proteins.*

**Supplementary Information:**

The online version contains supplementary material available at 10.1007/s00253-023-12743-6.

## Introduction

*Staphylococcus aureus* is a commensal microorganism inhabiting the skin, nares, and mucosae of livestock and humans. This bacterium can occasionally subvert the immune system and cause acute and chronic infections, ranging from skin wounds, pneumonia, and endocarditis to bloodstream invasion followed by sepsis (Kümmel et al. 2016; Sakr et al. 2018). Along with *Enterococcus faecium*, *Klebsiella pneumoniae*, *Acinetobacter baumannii*, *Pseudomonas aeruginosa*, and *Enterobacter* spp., *S*. *aureus* is part of the ESKAPE group, which includes opportunistic pathogens most commonly responsible for causing nosocomial infections (Maciejewska et al. 2018; Ma et al. 2020).

Besides its clinical relevance, *S*. *aureus* is also of major concern in agriculture, and it stands as one of the main causes of bovine mastitis in dairy cows, a multifactorial inflammatory disease where the animal, environment, udder microbiome, and pathogen-associated factors are key components of this complex disease (Haag et al. 2019; da Silva et al. 2020; Campos et al. 2022; Winther et al. 2022). *S*. *aureus* can encode a vast arsenal of virulence factors that are commonly correlated with the development of severe infections (Gill et al. 2011). Its virulence is based on the production of adhesion proteins and toxins, which avoid the capture by phagocytes and other immune system cells, as well as its common biofilm capability (Chung and Toh 2014; Kaźmierczak et al. 2014; Sakr et al. 2018).

The global burden of multidrug-resistant strains of *S*. *aureus*, such as methicillin-resistant *S*. *aureus* (MRSA), has been considered a strong public health concern and a priority pathogen by the World Health Organization (Sulis et al. 2022). Hence, the search for an effective and widely accessible new generation of antimicrobial compounds capable of controlling the growth of such pathogenic bacteria represents an urgent demand and deserves global attention (Bhandari and Suresh 2022).

Bacteriophages, also known as phages, are viruses that infect bacteria. Despite being considered the most abundant entities on Earth (about 10^31^ particles around the biosphere), there is still much to discover about phage biology (Hendrix 2002). “Jumbo phage” is a relatively recent classification of phages that possess exceptionally large genomes (greater than 200 kb in length) with unusual properties, such as a non-modular genome structure, presence of several genes that encode complex proteins (e.g., different types of DNA polymerases, multi-subunit RNA polymerases, RNA ligase and enzymes for NAD^+^ synthesis), and an even greater number of genes with unknown function (Yuan and Gao 2017). Currently, only six viruses classified as *Staphylococcus* jumbo bacteriophages have been meticulously characterized (Uchiyama et al. 2014; Korn et al. 2021; Lee et al. 2021; Zhang et al. 2022).

Jumbo phages and their unique genomic traits make them more independent of the host machine than the other phages and with still uncovered biological features of biotechnological interest (Iyer et al. 2021; Korn et al. 2021; Nazir et al. 2021). For instance, enzymes such as endolysins, virion-associated lysins, and polysaccharide depolymerases—phage-encoded proteins that act cleaving peptidoglycan bonds, or, in the case of depolymerases, the biofilm matrix—have been considered promising alternatives to tackle antimicrobial resistance, along with phage preparations (Fischetti 2018; Maciejewska et al. 2018; Melo et al. 2018; Akturk et al. 2019). Herein, the aim of this work is (i) to biologically characterize the jumbo phage vB_SauM-UFV_DC4 and (ii) to provide a genomic comparison with other jumbo phages that infect *S*. *aureus* to identify genomic traits of interest, such as lytic enzymes.

## Materials and methods

### Bacterial strains and culture conditions

In total, 26 isolates of *Staphylococcus aureus* obtained from different ecological niches (human hospital, *n* = 4; veterinary hospital, *n* = 14; meat products, *n* = 4; environmental, *n* = 4) were used in this study (Table S1). All strains were routinely cultivated on BHI broth (Kasvi, Curitiba, Brazil) with moderate shaking (100 rpm, Tecnal TE-420, Brazil) at 37 °C. In all experiments with phages, the temperature was set at 30 °C. The strain *S*. *aureus* 3059 was deposited in the Collection of Microorganisms, DNA and Cells of Universidade Federal de Minas Gerais (UFMG) belonging to the World Data Centre for Microorganisms under the accession number UFMG-CM-B275.

### vB_SauM-UFV_DC4 isolation and physiological features

The virus DC4 was isolated as described by da Silva Duarte et al. (2020). In summary, samples taken from wastewater of a dairy farm located at the Departamento de Zootecnia, Universidade Federal de Viçosa (DZO/UFV) at Viçosa, Minas Gerais, Brazil, were used for viral isolation. Samples were centrifuged and diluted (1:4) in SM buffer. The viral suspension was double-filtered through pore-size PES membranes (0.45 and 0.22 µm) and added to an early log-phase culture of *S*. *aureus* 3059 grown in BHI broth. The mixture was incubated at 30 °C for phage attachment and then plated using the standard soft agar overlay technique. The bacteriophage phage vB_SauM-UFV_DC4 is available upon request and can be obtained by contacting the corresponding author.

### One-step growth curve

The one-step growth curve protocol was performed as described by Kropinski (2018), with some adaptations. Briefly, the virus DC4 was added to a bacterial culture (*S*. *aureus* 3059) at the beginning of the exponential growth phase (OD_600nm_ 0.2; 1.6 × 10^8^ CFU/mL), at an MOI of 0.00001. After 10 min of incubation at 30 °C with moderate agitation (100 rpm) aiming for phage adsorption, the moisture was centrifuged at 10,000 g for 10 min at room temperature (Eppendorf 5804 Benchtop Centrifuge), the supernatant discarded, and the pellet was resuspended in 30 mL of fresh and pre-warmed BHI medium. Afterward, the mixture was incubated at 30 °C, setting-up an agitation of 100 rpm, for 5 h. Viral titration was carried out through the double-layer agar assay, where 100 µL of the sample was collected at different time points with an interval of 20 min and immediately plated. The latent period (delay between phage adsorption and phage particle release) was calculated by measuring the time interval between the phage infection and the beginning of the phage titer increase. The burst size (number of phages released after each infection round) was calculated by dividing the number of phages at the first peak of the one-step growth curve graphic, by the titer of phages at time zero of the experiment. Data obtained over three independent biological replicates, with three technical replicates for each, were used for graph construction and latent period/burst size determination.

### Host range determination and efficiency of plating (EOP)

In total, 26 isolates of *S*. *aureus* were used to evaluate the host range of the DC4 phage. Thus, 100 µL of 1 × 10^8^ PFU/mL phage suspension was used to perform ten-fold serial dilutions that were then plated with the different hosts, using the double-agar layer assay. The BHI top agar was made using 0.35% of agar, and the plates were incubated at 30 °C, overnight. After determining the phage titer in the different hosts, the EOP was calculated using the titer obtained in the strain *S*. *aureus* 3059 (host of isolation) as reference. The average EOP value for the combination DC4-bacterium was classified according to the criteria established by Mirzaei and Nilsson (2015). Data obtained over three independent biological replicates, with three technical replicates for each, were used for EOP calculation.

### Phage stability at different temperatures and pH

The stability protocols were performed as described by Jurczak-Kurek et al. (2016). In brief, to evaluate the stability of viral particles at different thermal conditions, 100 µL of 1 × 10^8^ PFU/mL DC4 phage suspension was diluted in 900 µL of BHI medium. The microcentrifuge tubes were kept, in triplicate, at − 20 °C for 12 h, 40 °C and 62 °C for 2 h, and 95 °C for 5 min. Tubes kept at room temperature for 2 h were used as control. To evaluate the stability of viral particles in different pH values, 100 µL of the DC4 phage suspension was diluted in 900 µL of BHI medium with different pH values (2, 4, 10, and 12). Phages diluted in BHI medium with pH 7 were used as control. The tubes were kept at room temperature for 2 h, in triplicate. After the respective treatments, the quantification of viable viral particles was performed using the double-agar layer assay. The phage titer after the treatments was compared to the initial titer.

### Statistical analysis

To evaluate the significance of the DC4 titers after the stability treatments, a one-way ANOVA and Dunnet’s post-hoc tests were performed (*p*-value < 0.05).

### vB_SauM-UFV_DC4 DNA extraction and sequencing

For viral DNA extraction, the protocol described by Amend et al. (2023) was followed and adapted with the necessary modifications. Briefly, a high viral titer (~ 10^10^ PFU/mL) was obtained after phage propagation via plate lysate and viral concentration from the bacterial lysate by using Vivaspin® 20 centrifugal concentrators with a molecular size cut-off of 100 kDa (GE Healthcare Life Sciences, UK). To the viral suspension (1 mL), 0.0125 M MgCl_2_ was added and mixed gently. Afterward, the lysate-MgCl_2_ was treated with 0.8 µL of DNase I (2000 U/mL) (Sigma-Aldrich, USA), and incubated at 37 °C for 30 min. To this mixture, 40 µL of 0.5 M EDTA, 5 µL of Proteinase K (10 mg/mL), and 50 µL of 10% SDS were added, vigorously vortexed, and incubated at 55 °C for 60 min. After this period, 500 µL of the mixture was transferred to 1.5-mL microcentrifuge tubes previously filled with the same volume of phenol:chloroform:isoamyl-alcohol (PCI) (25:24:1) and centrifuged (5 min at room temperature at 13,000 rpm, Eppendorf 5415C Centrifuge). The top aqueous phase layer was collected and transferred to a new 1.5-mL microcentrifuge tube with 500 µL of chloroform and a new centrifugation was conducted (5 min at room temperature at 13,000 rpm). To the top aqueous phase obtained from the previous step, DNA was precipitated by adding 50 µL of 3 M sodium acetate solution and 1 mL of 95% (v/v) ethanol. The sample was incubated for 5 min on ice and centrifuged at room temperature for 10 min at 13,000 rpm. The DNA pellet was washed with 500 µL of 70% (v/v) ethanol and finally in SpeedVac prior to resuspension in 50 µl of DNase/RNase-free distilled water. The purity and quality of the DNA were checked by electrophoresis on 0.8% (p/v) agarose gel, and DNA concentration was estimated by measuring the absorbance at 260 nm by using NanoDrop 2000 (Thermo Fisher, USA).

Viral DNA was sent to the Molecular Research DNA (Shallowater, TX, USA; mrdna.com). Sequencing was conducted with the Illumina MiSeq platform using PE reads (2 X 150 bp) and Nextera library preparation.

### Genomic analysis

The raw sequence data was evaluated and assembled de novo using the software SeqMan NGen 15 (DNAStar, Madison, WI, USA). Afterward, the contigs were inspected for internal errors, end trimmed, and reassembled using SeqManPro15. The obtained consensus sequence was annotated using Prokka (Seemann 2014). The sequence was further manually inspected for potential alternative start codons or the presence of non-annotated CDSs. The genome was then manually curated using Geneious 2022.0.2 (Biomatters Ltd.). BLASTX (Altschul 1997) was used to check for missing genes in intergenic regions. Encoded proteins were queried against protein sequences in BLASTP (Altschul 1997), PFAM (Finn et al. 2016), InterPro (Paysan-Lafosse et al. 2023), and HHpred (Söding et al. 2005) for homology search, protein families, signatures, and structure prediction. The potential coding sequences (CDSs) were also searched against Hidden Markov model profiles downloaded from the prokaryotic Virus Orthologous Groups (pVOGs) database (Grazziotin et al. 2017) using hmmscan (Eddy 2011). Protein sequences were further analyzed with TMHMM (Krogh et al. 2001), Phobius (Käll et al. 2007), and OCTOPUS (Viklund and Elofsson 2008) to predict transmembrane domains and SignalP (Petersen et al. 2011) and SPOCTOPUS (Viklund et al. 2008) to predict signal peptide cleavage sites. Intron sequences were predicted with Rfam (Kalvari et al. 2021).

The DC4 lifecycle (i.e., virulent, temperate, or chronic) was predicted by adopting a combination of Phage AI (machine learning models trained on 4694 manually selected bacteriophages from different species and families) (Tynecki et al. 2020) and BACPHLIP (searching for a particular set of temperate-specific protein domains) (Hockenberry and Wilke 2021). The proportion of non-coding ORFs present in DC4 was estimated using the Coding Potential Calculator 2 (CPC2) web server (Kang et al. 2017). Genes encoding tRNA were identified by using tRNAscan-SE (Lowe and Chan 2016) and ARAGORN (Laslett and Canback 2004). ResFinder 4.1 (Bortolaia et al. 2020) and HostPhinder 1.1 (Villarroel et al. 2016) were used to identify, respectively, the presence of acquired resistance genes (threshold for identity: 90%; minimum length: 60%; selected species: *Staphylococcus aureus*) and the in silico spectrum of phage hosts. Codon usage of DC4 and its hosts was calculated with SMS (sequence manipulation suite) taking into consideration the bacterial genetic code (Stothard 2000).

For phylogenomic purposes, phage genomes were downloaded and filtered from the National Center for Biotechnology Information (NCBI: txid10239) using inphared (INfrastructure for a PHAge REference Database, release 3 Oct 2021) (Cook et al. 2021). After a manual inspection, 83 whole-genome sequences (74 *Staphylococcus* phages and 9 *Bacillus* phages) were chosen based on their families (*Myoviridae*, *Rountreeviridae*, and *Siphoviridae*) and genome sizes (16.8 to 497.5 kb) (Table S2). At the time of selecting and filtering phages, tailed bacteriophages were still classified inside the former order *Caudovirales* (Turner et al. 2021). Therefore, the classical families *Myoviridae* and *Siphoviridae* were considered and mentioned throughout the text except when stated otherwise. Their relevant features were retrieved, and accession numbers were forwarded to VICTOR (Virus Classification and Tree Building Online Resource) (Meier-Kolthoff and Göker 2017) setting up amino acid as data type. VICTOR’s phylogenetic tree was annotated using IToL v6 (Interactive Tree of Life, version 6) (Letunic and Bork 2021). For taxonomic purposes, VIRIDIC (Moraru et al. 2020) and ViPTree (Nishimura et al. 2017) were adopted. The DC4 linear genomic map was constructed by using the ViPTree tool “genomic alignment view” and ORFs manually colored according to their function with INKSCAPE (version 1.2.2). Lastly, the R package corrplot was used for correlation analysis using the GC content and genome size as factors.

For comparative genomics, a pairwise comparison of all the *Staphylococcus* viruses’ genomes previously selected was carried out with the fastANI v1.3 algorithm (Jain et al. 2018) and based on the average nucleotide identity (ANI) method. Euclidean distances were computed among ANI results for clustering. progressiveMauve (Darling et al. 2010) within Geneious Prime 2022.0.2 was used to align and compute locally collinear blocks (LCBs) between PALS2 and DC4 genomes using default settings (match seed weight: 15; minimum LCB score: 30,000). Lastly, comparison and annotation of orthologous gene clusters among the six *S*. *aureus* jumbo phages (PALS2, SA1, Machias, Madawaska, MarsHill, and DC4) was conducted with OrthoVenn2 (E-value: 1e-2; Inflation value: 1.5) (Xu et al. 2019). BLAST Ring Image Generator (BRIG, version 0.95) was used to display circular comparisons among *S*. *aureus* jumbo genomes (Alikhan et al. 2011). The prediction of lytic and biofilm-degrading enzymes was based on the identification of specific and conserved domains such as CHAP (cysteine, histidine-dependent amidohydrolases/peptidases), SH3 (Src homology 3), and peptidoglycan binding domain (PGBD) using the InterPro protein families and domain database (Blum et al. 2021). Selected proteins were concatenated and aligned using Geneious Prime version 2022.0.2 with the BLOSUM62 cost matrix.

The complete genome sequence of vB_SauM-UFV_DC4 was deposited in GenBank under the accession number MZ779063.

### Phage purification and proteomic analysis

After virus propagation and concentration (item 2.3), the viral suspension was purified by a three-step protocol using ion exchange and desalting columns in a chromatography system (ÄKTAprime plus, GE Healthcare Life Sciences, Uppsala, Sweden) as adopted previously for virus vB_EcoM-UFV13 (da Silva Duarte et al. 2018b; da Silva Duarte et al. 2018a). Salts present in the sample were removed (step 1) using the HiTrap Desalting prepacked column (GE Healthcare Life Sciences, Uppsala, Sweden). The first two peaks were collected and purified using an anion exchange chromatography column (step 2), with specific fractions (7 to 12) carried out to the third and final step, a new desalting procedure. For the anion exchange column, start (20 mM Tris-HC, pH 8.0) and elution buffers (20 mM Tris-HC, 1 M NaCl, pH 8.0) were used, whereas in desalting steps, a phosphate buffer (20 mM sodium phosphate, 0.15 M NaCl, pH 7.0) was prepared. A flow rate of 5 mL.min^−1^ was adopted for both columns. The final viral titer was carried out by the double-agar overlay method using *S*. *aureus* 3059 as the host strain. Plates were incubated at 30 °C overnight.

After phage purification, viral particles were lyophilized and sent to GenOne (Rio de Janeiro, RJ, Brazil) for DC4 structural proteins extraction, quantification, and enzymatic digestion using trypsin. Peptides were injected in LCMS-NanoUltra-HPLC ultimate 3000 and Quadropole-Orbitrap QExactive Plus Gradient. Finally, bioinformatic analysis was carried out with Proteome Discoverer (Thermo Fisher Scientific, Waltham, MA, USA) software for protein identification using DC4-annotated proteins as a reference.

## Results

### vB_SauM-UFV_DC4 physiological features and host range analysis

**Fig. 1:**
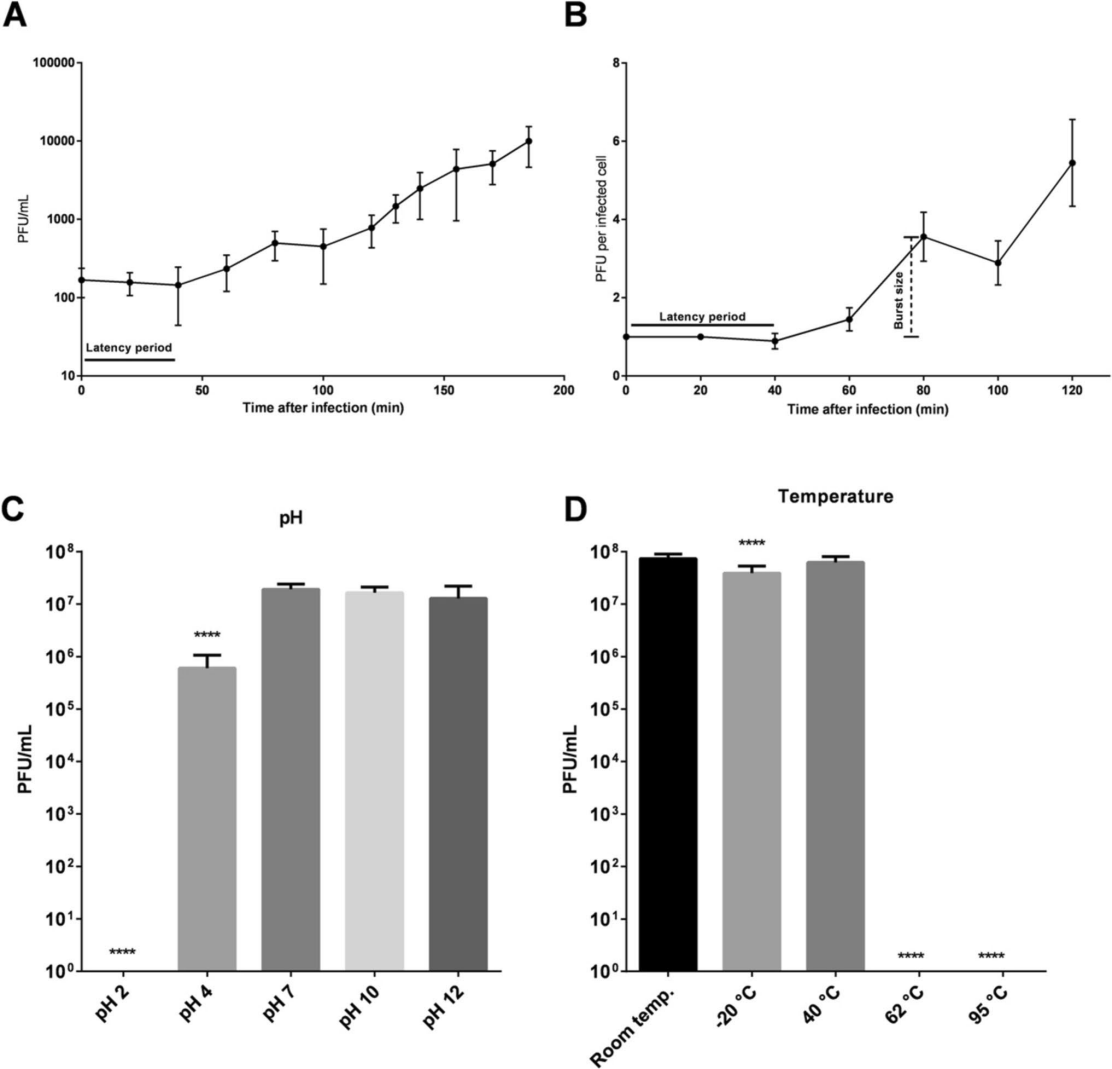
One-step growth curve and physical/chemical stability assays. **A** The data shown for DC4 one-step growth curve are the mean values from three independent measurements performed in triplicate. For a better plotting, only data collected until the first 200 min of experiment are shown and expressed in PFU per infected cell (**B**). The virus DC4 demonstrated a latent period of 40 min followed by a small burst of progeny (3 PFUs per infected cell). The stability of vB_SauM-UFV_DC4 under different temperatures (**C**) and pH (**D**) was evaluated. *p < 0.05 (*n* = 9). The error bars represent the standard deviations

One-step growth curve for the phage DC4 with the host strain *S*. *aureus* 3059 showed a latent period of 40 min followed by a small burst of 3.4 PFUs/infected cell (Fig. 1A, B). The virus DC4 displayed multiple small bursts after two additional lag periods of 40 min (t80–t120 = 1.5 PFU/infected cell; t120–t160 = 5.7 PFUs/infected cell). The physical and chemical stability of phage DC4 was determined to provide more information regarding phage storage and potential applications at different conditions (Fig. 1C, D). More than 94% of DC4 particles survived at pH values ranging from 7 to 12, and 71.4% survived pH 4; however, DC4 could not survive pH 2. DC4 was stable at temperatures ranging from − 20 to 40 °C but not at 62 and 95 °C. The lytic activity of phage DC4 against 26 strains of *Staphylococcus aureus* is shown in Table S1. Phage DC4 could infect 11 out of 26 *S*. *aureus* strains (10 from a veterinary hospital and 1 from a hospital for humans). The relative EOP of phage DC4 was also measured for 11 different strains, and it was found that DC4 can efficiently propagate in nine isolates (Table 1).

**Table 1:** *S. aureus* isolates obtained from a veterinary hospital tested as potential hosts of DC4 phage

Host	Infection	Average titer	Efficiency of plating (EOP)
S. aureus 3059*	+	2,97E + 08	
*S. aureus* 3907	+	4,03E + 08	High
*S. aureus* 1334	+	2,80E + 08	High
*S. aureus* 222	+	5,87E + 08	High
*S. aureus* 607 HV	+	2,30E + 07	Low
*S. aureus* 574 HV	+	4,07E + 08	High
*S. aureus* O46	+	8,63E + 08	High
*S. aureus* 3.2	+	1,27E + 08	Medium
*S. aureus* 32/2	+	1,60E + 08	High
*S. aureus* 4182	+	2,13E + 08	High
*S. aureus* 4081	+	3E + 08	High

High, efficiency of plating assay using hosts that presented a ratio of 0.5 or more; medium, efficiency of plating assay using hosts that presented a ratio between 0.5 and 0.1; low, efficiency of plating assay using hosts that presented a ratio smaller than 0.1

^*^Host routinely used for DC4 propagation

### Genomic features of vB_SauM-UFV_DC4

**Fig. 2:**
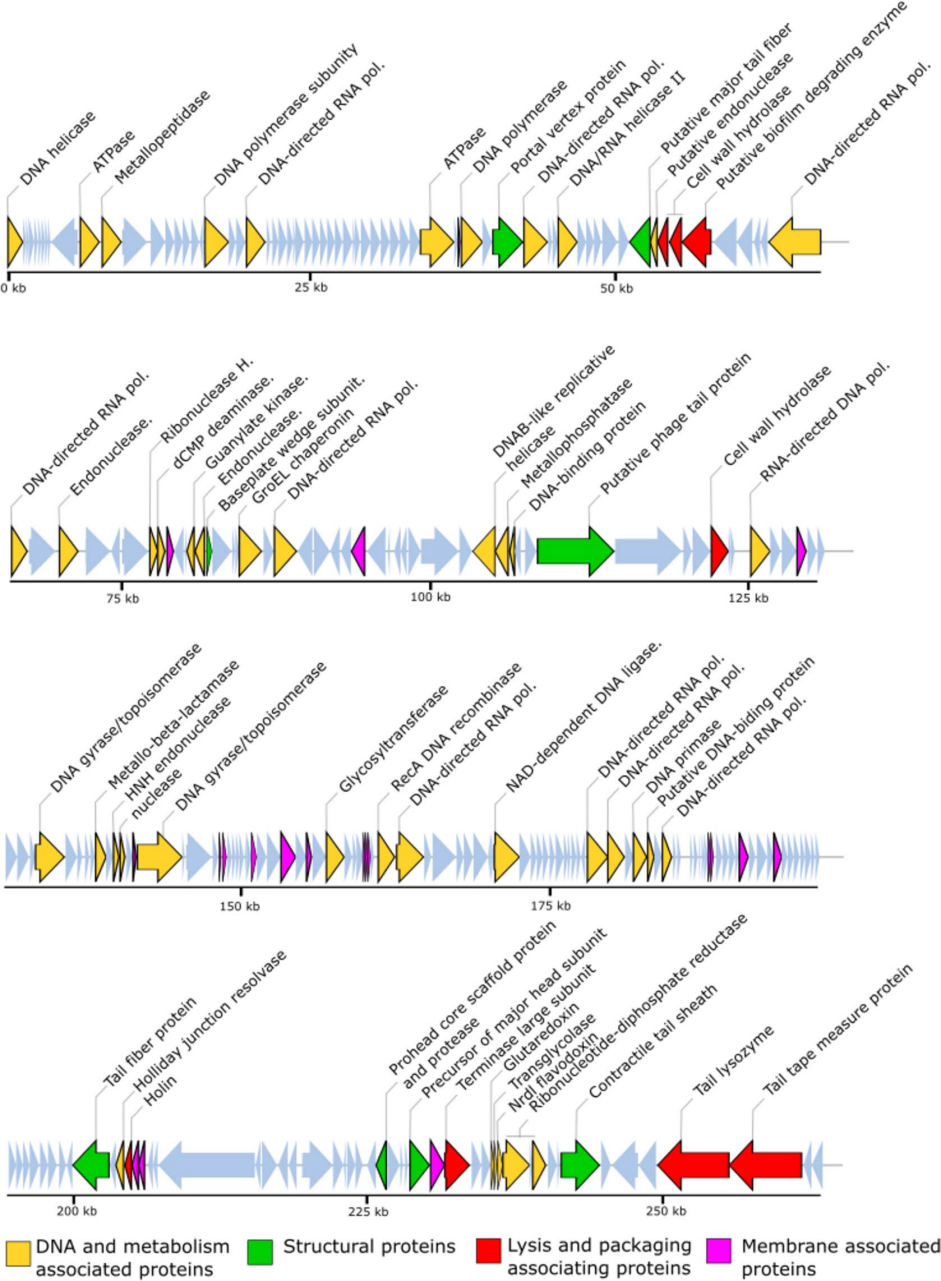
The linear genome map of phage DC4 showing position of CDSs. The CDSs are colored according to their functional category

The phage DC4 genome is a 263,185 bp contiguous sequence of linear, double-stranded DNA with an overall G + C content of 25% (Fig. 2; Fig. S1) and encodes 263 predicted CDSs. The correlation analysis between large genomes and low GC content relationship carried out with *Staphylococcus* viruses revealed a significant and moderate negative correlation between genome size and GC content (Pearson *R* value =  − 0.59, *p* < 0.001; Table S3). The gene-coding potential of the global genome is 91.9% with only eight overlapping genes and displays. Among all the CDSs, 207 initiated translation with an ATG start codon, and most genes (79.8%) are transcribed from the positive strand. The DC4 genome also encodes 1 tRNA (Phe-tRNA: genome coordinates − 52,143–52,236 kb) and has a predicted pseudo-tRNA (Leu-tRNA: 72,405–72,475 kb). Following gene inspection and annotation with different approaches, 54 genes received a final functional assignment (Table 2), 185 were assigned as “hypothetical protein,” and 24 were predicted as “hypothetical membrane protein” (proteins with predicted transmembrane domains). No antimicrobial resistance genes were identified, as well as genes with predicted lysogeny functions (e.g., integrase, transposase, and excisionase). Indeed, BACPHLIP predicted that DC4 has a lower probability of being a temperate phage (23.75%). On the contrary, the tool PhageAI, which infers bacteriophage lifestyle with approximately 98% of accuracy, has predicted DC4 as a temperate virus. Considering the prediction of mobile self-splicing elements, Rfam did not identify any intronic sequences in the DC4 genome. However, after protein screening using InterPro, five sequences stand out and the C-terminal RSV-like homing endonuclease domain (Gene3D: G3DSA:3.40.960.10). The UFVDC4_00129 gene encodes the putative HNH homing endonuclease (Pfam: PF13392). Only one intein was observed, and it is present within the protein-coding gene *gyrA* (DNA gyrase subunit A). An endonuclease domain LAGLIDADG_3 (Pfam: PF14528, IPR004860) was also identified.

**Table 2:** vB_SauM-UFV_DC4 functional genomic annotation. The final function was determined based on the combination of different approaches. The 185 ORFs assigned as “hypothetical protein,” and the 24 ORFs predicted as “hypothetical membrane protein” are not included

Locus	Min	Max	Strand	NN	AA	MW	I.P	Blast	Function
UFVDC4_00001	186	1394	+	1209	402	46.02	7.64	Exodeoxyribonuclease V subunit alpha	ATP-dependent RecD-like DNA helicase
UFVDC4_00011	6096	7646	+	1551	516	59.61	4.83	AAA family ATPase	AAA family ATPase
UFVDC4_00012	7717	9648	+	1932	643	74.33	4.40	Hypothetical protein	Metallopeptidase
UFVDC4_00020	16296	18230	+	1935	644	74.94	5.45	DNA-polymerase catalytic subunit	DNA-polymerase subunit
UFVDC4_00023	19699	21210	+	1512	503	57.84	4.62	DNA-directed RNA polymerase beta' subunit	DNA-directed RNA polymerase beta' subunit
UFVDC4_00043	33937	36672	+	2736	911	106.36	4.58	SbcC ATPase	ATPase
UFVDC4_00046	37289	39010	+	1722	573	66.26	4.94	DNA polymerase	DNA polymerase
UFVDC4_00048	39857	42298	+	2442	813	92.80	4.35	Portal vertex protein	Portal vertex protein
UFVDC4_00049	42399	44312	+	1914	637	74.29	4.57	DNA-directed RNA polymerase beta subunit	DNA-directed RNA polymerase beta' subunit
UFVDC4_00052	45209	46756	+	1548	515	60.71	9.40	DNA/RNA helicase of superfamily II	DNA/RNA helicase II
UFVDC4_00059	51043	52713	−	1671	556	61.68	4.70	Ig-like domain-containing protein	Ig-like domain-containing protein
UFVDC4_00060	53361	54158	−	798	265	29.39	10.55	Endolysin	Cell wall hydrolase
UFVDC4_00061	54324	55274	−	951	316	34.92	9.77	N-acetylmuramoyl-L-alanine amidase	Cell wall hydrolase
UFVDC4_00067	62429	66682	−	4254	1417	162.50	5.09	DNA-directed RNA polymerase beta subunit	DNA-directed RNA polymerase beta subunit
UFVDC4_00068	67143	68369	+	1227	408	45.74	5.40	DNA-directed RNA polymerase beta subunit	DNA-directed RNA polymerase beta' subunit
UFVDC4_00070	70912	72363	+	1452	483	58.51	9.63	Hef-like homing endonuclease	Hef-like homing endonuclease
UFVDC4_00074	78011	78631	+	621	206	24.17	8.98	Ribonuclease H	Ribonuclease H
UFVDC4_00075	78632	79186	+	555	184	20.97	4.51	dCMP deaminase	dCMP deaminase
UFVDC4_00080	80925	81485	−	561	186	21.69	5.46	Guanylate kinase	Guanylate kinase
UFVDC4_00081	81562	82293	−	732	243	29.06	9.80	Endonuclease fused to N-terminal Zn finger domain	Endonuclease
UFVDC4_00082	82473	82874	+	402	133	14.94	4.52	Base plate wedge subunit	Base plate wedge subunit
UFVDC4_00086	85037	86779	+	1743	580	65.36	4.65	Chaperonin GroEL	GroEL chaperonin
UFVDC4_00088	87799	89532	+	1734	577	68.22	4.89	DNA-directed RNA polymerase subunit	DNA-directed RNA polymerase subunit
UFVDC4_00101	103382	105124	−	1743	580	67.00	4.65	DnaB-like replicative helicase	DnaB-like replicative helicase
UFVDC4_00102	105149	106291	−	1143	380	44.79	4.33	Metallophosphatase	Metallophosphatase
UFVDC4_00103	106284	106667	−	384	127	14.93	4.38	Hypothetical protein	DNA-binding protein
UFVDC4_00110	122138	123463	+	1326	441	51.32	6.96	N-acetylmuramoyl-L-alanine amidase	Cell wall hydrolase
UFVDC4_00113	125252	126754	+	1503	500	58.89	9.48	RNA-directed DNA polymerase	RNA-directed DNA polymerase
UFVDC4_00122	133435	135717	+	2283	760	86.70	5.09	DNA gyrase/topoisomerase IV, subunit B	DNA gyrase/topoisomerase IV, subunit B
UFVDC4_00127	138206	139033	+	828	275	32.39	5.59	Metallo-beta-lactamase superfamily protein	Metallo-beta-lactamase
UFVDC4_00129	139623	140054	+	432	143	17.13	9.98	HNH endonuclease	HNH endonuclease
UFVDC4_00130	140147	140518	+	372	123	14.35	4.67	Nuclease	Nuclease
UFVDC4_00132	141522	145076	+	3555	1184	136.22	7.59	DNA gyrase/topoisomerase IV, subunit A	DNA gyrase/topoisomerase IV, subunit A
UFVDC4_00145	151812	152912	+	1101	366	42.62	4.52	Toxic anion resistance protein	Toxic anion resistance protein
UFVDC4_00150	156540	157946	+	1407	468	54.25	9.59	Glycosyltransferase	Glycosyltransferase
UFVDC4_00158	160655	161956	+	1302	433	48.64	4.73	RecA-like DNA recombinase	RecA DNA recombinase
UFVDC4_00159	162110	164251	+	2142	713	82.82	5.53	DNA-directed RNA polymerase beta subunit	DNA-directed RNA polymerase beta subunit
UFVDC4_00166	169875	171863	+	1989	662	75.92	4.55	NAD-dependent DNA-ligase	NAD-dependent DNA-ligase
UFVDC4_00178	177264	178886	+	1623	540	63.15	4.93	DNA-directed RNA polymerase beta subunit	DNA-directed RNA polymerase beta subunit
UFVDC4_00179	178886	180196	+	1311	436	49.88	8.46	DNA-directed RNA polymerase beta' subunit	DNA-directed RNA polymerase beta' subunit
UFVDC4_00181	180916	181968	+	1053	350	41.43	8.91	DNA primase	DNA primase
UFVDC4_00220	201056	204052	−	2997	998	111.16	4.73	Host specificity protein	Tail fiber protein
UFVDC4_00222	204624	205250	−	627	208	25.14	9.70	Holliday junction resolvase	Holliday junction resolvase
UFVDC4_00223	205370	205909	−	540	179	19.46	4.09	Holin	Holin
UFVDC4_00239	226113	226955	−	843	280	32.07	5.79	Prohead core scaffold protein and protease	Prohead core scaffold protein and protease
UFVDC4_00242	228918	230522	+	1605	534	59.24	4.44	Precursor of major head subunit	Precursor of major head subunit
UFVDC4_00244	231796	233829	+	2034	677	78.39	8.57	Terminase large subunit	Terminase large subunit
UFVDC4_00247	235613	235852	+	240	79	8.75	4.46	Glutaredoxin	Glutaredoxin
UFVDC4_00249	236165	236569	+	405	134	15.21	4.96	NrdI flavodoxin	NrdI flavodoxin
UFVDC4_00250	236596	238797	+	2202	733	84.05	4.75	Ribonucleotide-diphosphate reductase subunit alpha	Ribonucleotide-diphosphate reductase alpha subunit
UFVDC4_00252	239057	240127	+	1071	356	41.27	4.37	Ribonucleotide-diphosphate reductase subunit Beta	Ribonucleotide-diphosphate reductase beta Subunit
UFVDC4_00255	241411	244563	+	3153	1050	118.88	4.51	Contractile tail sheath structural protein	Contractile tail sheath
UFVDC4_00259	249374	255262	−	5889	1962	211.94	9.63	Tail lysozyme	Tail lysozyme
UFVDC4_00260	255286	261258	−	5973	1990	216.57	10.15	Tail tape measure protein	Tail tape measure protein

*NN* nucleotide length, *AA* amino acid length, *I.P* isoelectric point, *CDS* coding sequences

### Functional features

At least nine multi-subunit DNA-dependent RNA polymerases (RNAPs) and a Sigma70 factor (UFVDC4_00065) were identified on phage DC4 genome. One of these subunits (UFVDC4_00067) was also identified packaged into the virion (vRNAP) along with a transcriptional factor (UFVDC4_00011) belonging to the AAA family ATPase. The virus also displays the four DNA-directed RNAP subunits with high similarities to those enzymes described in the PALS2 genome (Lee et al. 2021) (β: UFVDC4_00159, PALS2_067 – 99.72%; β′: UFVDC4_00179, PALS2_089 – 100%; ω: DC4_00013, PALS2_188 – 99.27%; δ: UFVDC4_00049, PALS2_228 – 98.12%). The annotation of the other five genes showed that ORFs 23 and 69 encode for β subunits and show high homology with those found in the *Staphylococcus* phage Madawaska (98.41 and 98.04%), while the genes UFVDC4_00067 and UFVDC4_00178 encode for β subunits and display high identity with coding sequences found in the *Staphylococcus* phage Madawaska (98.45%) and *Staphylococcus* phage MarsHill (98.52%), respectively. Lastly, UFVDC4_00087 was annotated as a non-viral RNA polymerase subunit (*Staphylococcus* phage MarsHill, 98.44%). Another noteworthy feature on DC4 genome is the presence of a predicted RNA-dependent DNA polymerase (reverse transcriptase) (Pfam: PF00078). This is a conserved protein among the *S*. *aureus* jumbo phages encompassed in this study sharing 84% of identity. In the case of DC4, this corresponds to locus tag UFVDC4_00113 (UAJ17040.1), a basic 58.9 kDa protein.

**Fig. 3:**
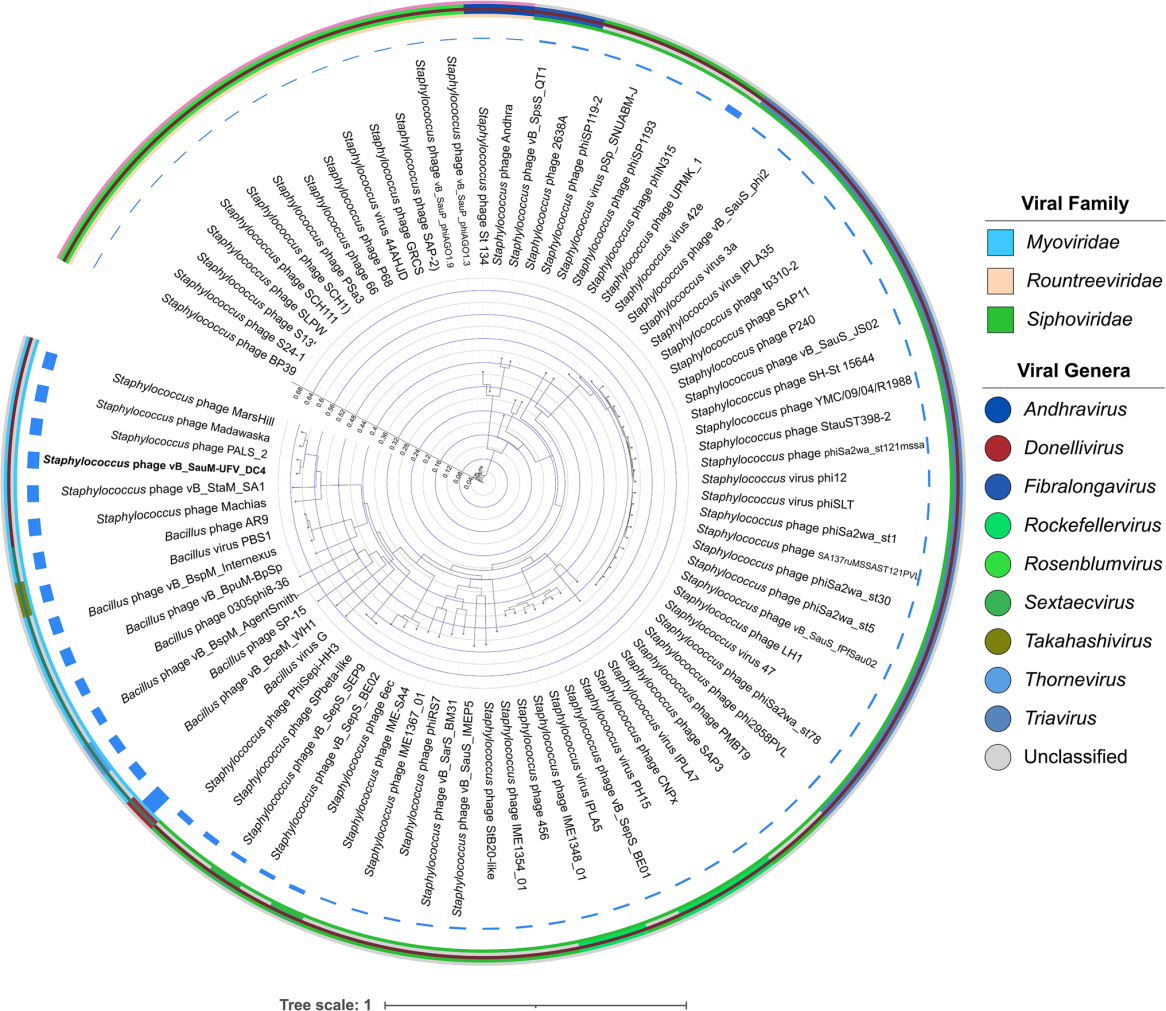
Interactive Tree Of Life (iTOL) was constructed based on VICTOR’s phylogenetic tree using 83 whole-genome sequences (74 *Staphylococcus* phages and nine *Bacillus* phages). Outer and inner rings show viral families and genera, respectively. The color saturation indicates the degree of difference in terms of genome size

In silico bacterial host prediction using HostPhinder revealed that DC4 might have several bacterial genera as hosts, including *Staphylococcus* and *Bacillus* (Table S2). The initial functional annotation at the protein level of the phage DC4 revealed a homology of 22% with the phages AR9 (KU878088) and PBS1 (MF360957) and 21% with the phage vB_BpuM-BpSp (KT895374), both infecting *Bacillus* sp. However, genomic comparison including 74 *Staphylococcus* phages and nine *Bacillus* jumbo phages showed that the phage DC4 is phylogenetically related to five recently reported *Staphylococcus* jumbo phages (Machias, MW349128; Madawaska, MW349129; MarsHill, MW248466; PALS2, MN091626; and vB_StaM_SA1, MW218148) (Fig. 3).

### Phylogenomic

**Fig. 4:**
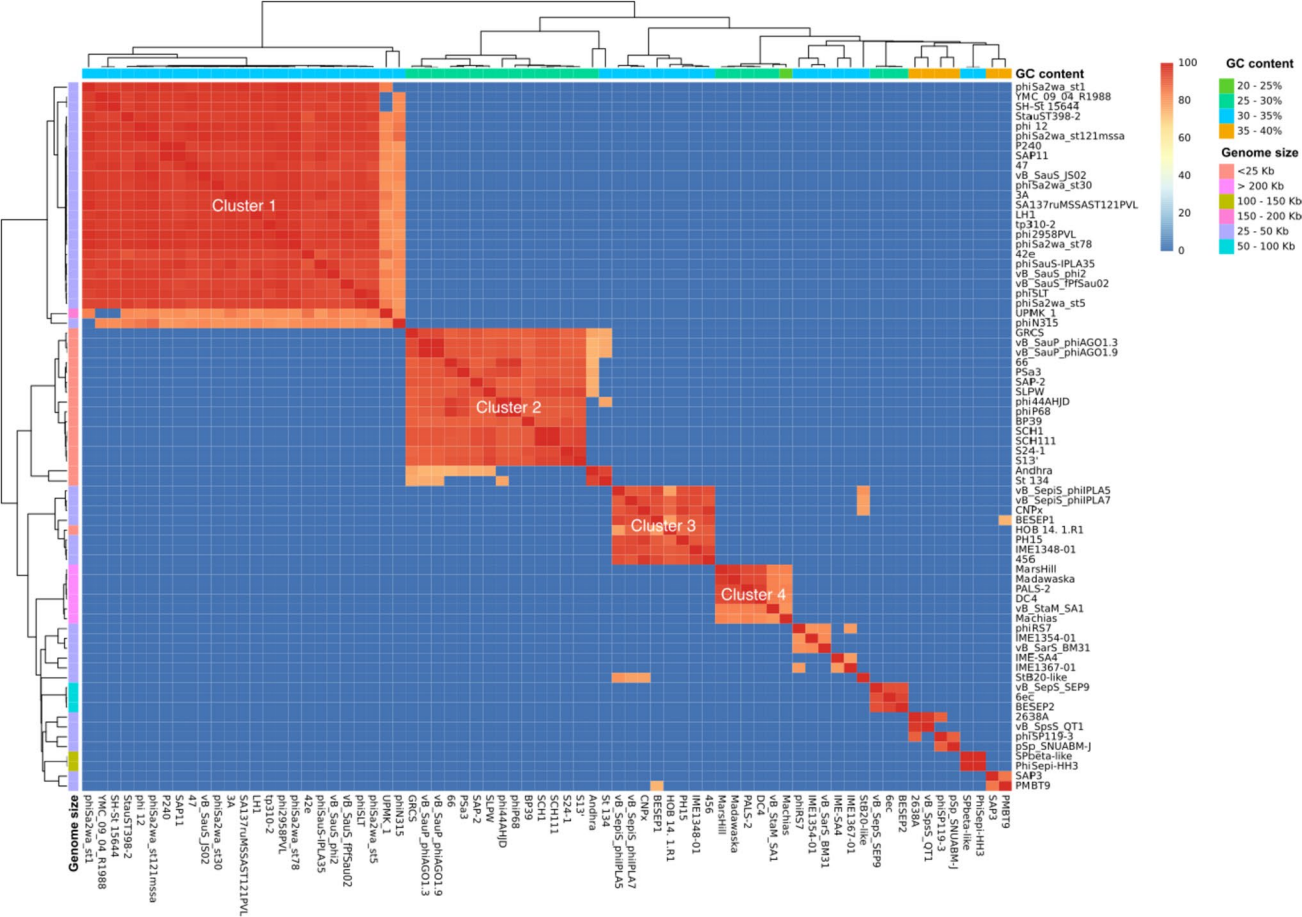
Heatmap of the average nucleotide identity (ANI) between the whole genome sequences of bacteriophages displaying *S*. *aureus* as host (*n* = 74)

**Fig. 5:**
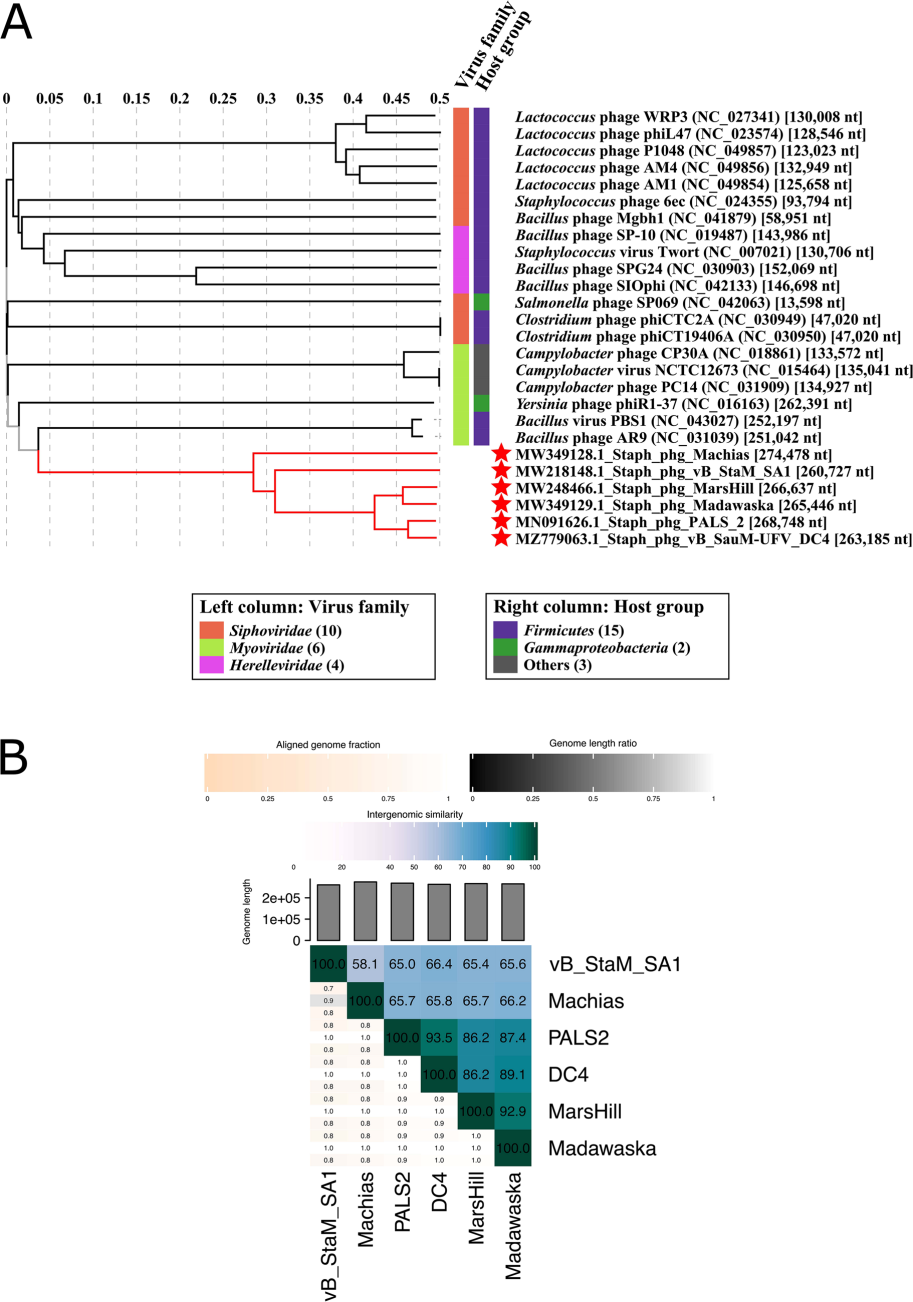
VIRIDIC (**A**) generated tree (BLASTN homology) of *S*. *aureus* jumbo phages (red stars) and unrelated viruses infecting the different hosts (phylum *Firmicutes* and class *Gammaproteobacteria*) shows that DC4 is one of four new species in a new genus. ViPTree (**B**) based on the proteome of *S*. *aureus* jumbo phages (TBLASTX homology) reveals that DC4 is probably part of a new family composed of two subfamilies and one genus

The whole-genome similarity among the DC4 and other *Staphylococcus* phages was estimated based on phylogenomic analysis. As depicted in Fig. 4, it is possible to identify at least four relevant clusters showing high ANI scores based on gene content. Interestingly, each cluster is characterized by a different range of GC content combined with genome size (cluster 1: 30–35% GC and 25–50 Kb; cluster 2: 25–30% GC and < 25 Kkb; cluster 3: 30–35% GC and 25–50 kb; cluster 4: 25–30% GC and > 200 kb). Overall, viruses belonging to the genera *Triavirus*, *Rosenblumvirus*, and *Rockefellervirus* were grouped in the cluster 1, cluster 2, and cluster 3, respectively. Specifically, regarding cluster 4, which includes only *Staphylococcus* jumbo phages, VIRIDIC and VIPtree analyses (Fig. 5) revealed that the DC4 genome is one of four new species in a new genus and shows high similarity with the phage PALS2 (Fig. S2). Based on the frequency of codon usage for the amino acid phenylalanine, DC4 and its hosts (*S*. *aureus* 2030RH1 and 3059) show very similar fraction values for the codons TTT (~ 0.69) and TTC (~ 0.31) (Fig. S3).

### Orthologous and vB_SauM-UFV_DC4 lytic proteins

The comparative analysis of orthologous genes among the six *Staphylococcus* jumbo phages revealed 303 clusters (133 orthologous clusters present in at least two viruses) and 170 single-copy gene clusters (Fig. S4). Investigating the biological processes of the biggest cluster of genes (172 genes), the most enriched gene ontology (GO) terms were related to DNA replication (GO:0006260) and metabolic process (GO:0008152), both crucial for the phage life cycle (Weigel and Seitz 2006). Lastly, three genes were not included in any cluster (singletons: DC4_00155, DC4_00190, and DC4_00218) for the phage DC4; all of them were annotated as hypothetical proteins.

**Fig. 6:**
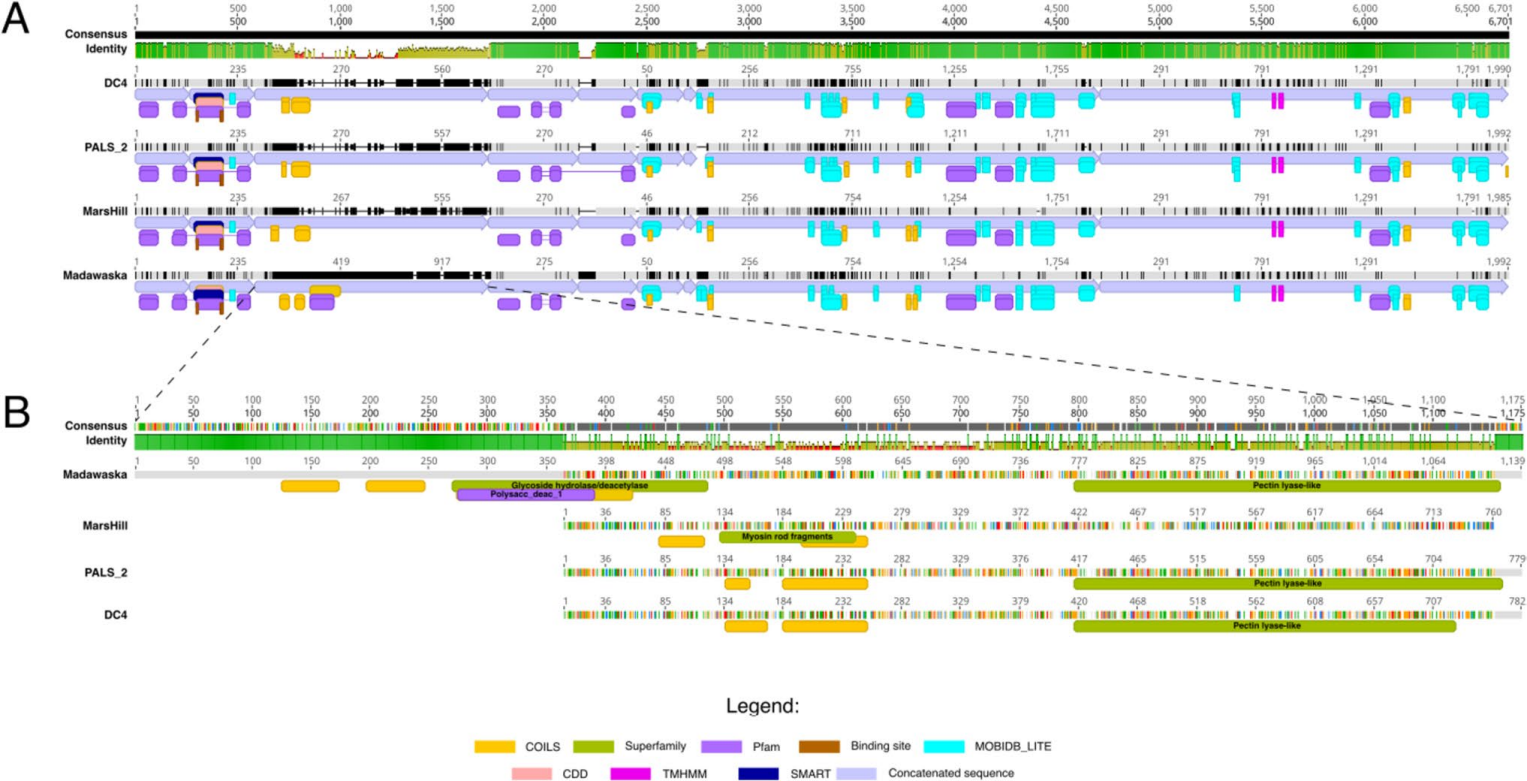
The nine putative lytic proteins (**A**) identified in the DC4 proteome were concatenated, aligned, and compared with their homologs found in the phages PALS2, Madawaska, and MarsHill. **B** Detailed analysis of the tail fiber protein with a pectin lyase domain among the four closely related *S*. *aureus* jumbo phages. Mean pairwise identity over all pairs in the column: green: 100% identity; greeny-brown: 30–100% identity; red: below 30% identity

Regarding the set of putative lytic enzymes encoded in the DC4 genome, nine proteins stand out and were further aligned and compared with their homolog proteins identified in the other four *S*. *aureus* jumbo phages (cluster 4, Figs. 4 and 6). Due to their low overall identity with DC4 proteins, phages vB_StaM_SA1 and Machias were not included in this analysis. Three out of nine proteins were classified as cell wall hydrolases and displayed CHAP + SH3 (DC4_0060, endolysin), amidase_2 + SH3 (DC4_0061, N-acetylmuramoyl-L-alanine amidase), and amidase_2 + PG_binding (DC4_00110, N-acetylmuramoyl-L-alanine amidase) domains. In two hypothetical proteins (DC4_00236 and DC4_00241), a PGBD domain was identified. The DC4_00248 encodes a putative transglycosylase IsaA. Lastly, three tail-associated enzymes were identified: DC4_0062 encodes a tail fiber with a pectin lyase–like domain, whereas a tail lysozyme (DC4_00259) and a tail tape measure protein with peptidase_M23 domain (DC4_00260) were also predicted. After concatenation and alignment, the identity matrix revealed that together the set of lytic enzymes predicted for the virus DC4 shares 97%, 88%, and 84% of identity with the phages PALS2, Madawaska, and MarsHill, respectively. As depicted in Fig. 5A, several amino acid substitutions can be observed comparing the four *S*. *aureus* jumbo isolates, and most of them are in domain regions, which might impact substrate recognition and binding. Between DC4 and PALS2, 169 residues were not identical, whereas, between DC4 and Madawaska/MarsHill, 774 and 844 differences were noticed, correspondingly. Noteworthy, a tail fiber protein containing a pectin lyase–like domain (DC4_0062) showed a very low conserved region when compared to the other viruses. A manual inspection revealed that in the phages DC4, PALS2, and MarsHill, this protein displays, on average, 773 aa, while the virus Madawaska encodes a protein with 1139 aa with an exclusive glycoside hydrolase/deacetylase domain (Fig. 5B).

With regard to the proteins identified as endolysins in this study, a high degree of conservation in terms of length and the primary sequence were observed among the 4 isolates. The viruses DC4 and PALS2 differ in 9 residues, whereas between DC4 and Madawaska/MarsHill, 11 and 45 differences were noticed, respectively.

### DC4 structural proteome

Phage structural proteome analysis revealed that 20 putative genes in the DC4 genome potentially code for virion proteins, such as portal vertex protein (ORF_0049), base plate wedge subunit (ORF_0083), tail fiber protein (ORF_0216), prohead core scaffold protein and protease (ORF_235), contractile tail sheath (ORF_251), precursor of major head subunit (ORF_0238), and tail tape measure protein (ORF_256).

To verify the structural and packaged proteins in the mature phage virion of DC4, purified phage particles were enzymatically treated and analyzed using LCMS. In total, 26 peptides were mapped to 15 proteins (Table 3), including the precursor of the major head subunit (UFVDC4_00242), tail fiber protein (UFVDC4_00062; UFVDC4_00220), and prohead core scaffold protein and protease (UFVDC4_00239). Among the other phage-coding proteins, six of them were identified as hypothetical proteins (UFVDC4_00056, UFVDC4_00234, UFVDC4_00107, UFVDC4_00019, UFVDC4_00134, and UFVDC4_00153), one as Ig-like domain-containing protein (UFVDC4_00059), and the other four proteins are GroEL (UFVDC4_00086), UvsX-like recombinase (UFVDC4_00158), a transcription factor belonging to AAA family ATPase (UFVDC4_00011) and RNAP β subunit (UFVDC4_00067). In addition, we also checked the peptides obtained for the protein GroEL against the genome of the host strains *S*. *aureus* 2030RH1 and 3059 to verify whether this protein is of bacterial origin; however, no similar pattern was observed in both strains.

**Table 3:** Characteristic of the DC4 structural proteins identified by LCMS

Locus	Annotation	Coverage	Peptides	PSMs	AAs	MW (kDa)	Calc. pI
UFVDC4_00242	Precursor of major head subunit	11.99	5	6	534	59.19	4.84
UFVDC4_00056	Hypothetical protein AR9_g161 (Bacillus phage AR9)	9.71	3	6	412	46.49	4.94
UFVDC4_00086	GroEL	11.03	5	5	580	65.31	5.03
UFVDC4_00234	Hypothetical protein	8.27	2	2	278	31.63	4.92
UFVDC4_00158	UvsX-like recombinase (Bacillus phage AR9)	3.70	1	1	433	48.60	5.11
UFVDC4_00011	Transcription factor (Bacillus virus PBS1)	1.75	1	1	514	59.32	5.24
UFVDC4_00062	Tail fiber protein (Staphylococcus phage IME1323_01)	1.15	1	1	782	87.60	4.74
UFVDC4_00239	Prohead core scaffold protein and protease	5.00	1	1	280	32.05	6
UFVDC4_00059	Ig-like domain-containing protein	1.44	1	1	556	61.64	5.07
UFVDC4_00220	Tail fibers	1.80	1	1	998	111.08	5.14
UFVDC4_00107	Hypothetical protein	1.20	1	1	1747	201.82	4.87
UFVDC4_00019	Hypothetical protein	24.18	1	1	91	10.87	10.2
UFVDC4_00067	DNA-directed RNA polymerase beta subunit	1.91	1	1	1417	162.38	5.48
UFVDC4_00134	Hypothetical virion structural protein	3.69	1	1	677	78.67	4.88
UFVDC4_00153	Hypothetical protein	10.00	1	1	180	21.58	9.01

*PSMs* peptide spectrum matches, *AAs* amino acids, *MW* molecular weight, *Coverage* percentage of the protein sequence covered by the peptides

## Discussion

Jumbo phages have attracted great attention over the last years due to their wide lytic activity (broad host range) and partial independence from the host enzymes in terms of gene expression (encoding their own RNA polymerases) (Yuan and Gao 2017; Sokolova et al. 2020). Furthermore, some isolates of jumbo viruses such as phiKZ-like phages infecting *Pseudomonas* can assemble a nucleoid structure during viral infection as an adaptive mechanism to evade bacterial defense systems like CRISPR-Cas restriction enzymes (Malone et al. 2019; Guan and Bondy-Denomy 2020). Overall, jumbo phages have been characterized as tailed bacteriophages (myovirus and siphovirus) with a genome size greater than 200 kb, have large particles, and show low similarity to those already described (Hendrix 2009). In terms of jumbo phages targeting *Staphylococcus*, genomic information of only six viruses can be found in public databases. The Machias (MW349128), Madawaska (MW349129), and MarsHill (MW248466) were isolated in the USA from swine barn; SA1 (accession no: MW218148) and bacteriophages S6 (accession no: LC680885) were obtained from sewage in China and Japan, respectively. The virus PALS2 (accession no: MN091626.1) was isolated from bird feces in South Korea. Among them, only PALS2 was completely characterized (Lee et al. 2021), which reflects the current scarcity of biological information about jumbo phages infecting *S*. *aureus*, one of the main important pathogens in human and veterinary medicine (Park and Ronholm 2021).

The physiological analysis showed that DC4 phage has similarities to other jumbo phages. When compared to the latest available data for *Staphylococcus* jumbo bacteriophages, DC4 shows the smallest burst size among PALS2 (12 PFUs/infected cell) and SA1 (140 PFU/infected cell), with an intermediate latency period (PALS2: 30 min; SA1: 55 min) The phage also presents some additional small burst events, which is a commonly observed feature for jumbo phages with small burst sizes (Shkoporov et al. 2018; Sharma et al. 2019; Lee et al. 2021). Despite data scarcity specifically related to jumbo phages stability, different studies have reported an apparent higher sensitivity of this group of phages to higher temperatures but not at basic pH values. The jumbo phage MIJ3 is stable at temperatures ranging from 4 to 60 °C, while more than 90% of MIJ3 phage survived at pH values ranging from pH 3 to ~ 10 (pH values above 10 were not tested) (Imam et al. 2019). The *Xanthomonas* jumbo phage LucasX remained viable up to 45 °C with a pH optimum of 7 but after 30 min at 50 °C, just a few particles were still viable. This phage also was completely inactivated on pH 3 and 11 (Marquioni et al. 2022). For the *Salmonella* jumbo phage pSal-SNUABM-04, viral particles were stable at 4–27 °C, whereas stability dropped at 37 °C within 2 h (Kwon et al. 2020). Our data shown that besides the DC4 phage present (as others jumbo phages) a relatively low resistance to high temperatures, it is still stable on pH 12, appearing to show greater resistance in this field than the phages discussed above. Also, the wide host range against staphylococci from the veterinary environment indicates that phage DC4 has the potential to be explored as an anti-staphylococcal agent for veterinary usage.

The GC content of the phage DC4 is within the range of other *Staphylococcus* jumbo phages (24.8–26.8%), which is significantly lower than that of their hosts (*S*. *aureus* 3059, 32.8%; *S*. *aureus* 2030RH1, 32.7%) and other phages with dsDNA genomes larger than 200 kb (Lavysh et al. 2016; Wojtus et al. 2019). The GC content of 25% is also only slightly higher than the *Staphylococcus* phage Machias (24.8%). As reported by Almpanis et al. (2018), bacteriophages with large genome sizes are more likely to present lower content of guanine and cytosine, whereas larger bacterial genomes tend to have higher GC content. Our results reinforce this idea, once a significant and moderate negative correlation between genome size and GC content was found among the *Staphylococcus* viruses evaluated on the correlation analysis. Although the evolutionary explanation for this phenomenon is still a matter of debate, environmental factors and physiological capabilities have a major influence in shaping the GC content of the phage genome, which can impact codon usage for protein synthesis (Almpanis et al. 2018). Phage DC4 also presents a considerably low gene density (genes per kilobase pair of nucleotide sequence) when compared to other jumbo phages (gene density of 1.5 ± 0.25) (Iyer et al. 2021), once its 263,185 bp genome encodes 263 predict CDSs.

Although the large size, the DC4 genome only encodes 1 tRNA and one predicted pseudo-tRNA. The low number of tRNAs encoded in DC4 genome is not exclusive for this virus but was also noticed or even not predicted for others *Staphylococcus* jumbo phages such as PALS2, S6 and Madawaska (1 tRNA-Asn), MarsHill (0 tRNA), Machias (2 pseudo tRNA-Asn), and vB_StaM_SA1 (tRNA-Ser). This result shows that these phages have underwent different selective pressures and retained different tRNAs based on host and virus codon usage. Moreover, it is quite intriguing that such “autonomous” phages, a common characteristic stated for jumbo phages, have a such low number of tRNA even when compared to other jumbo phages. In a comparative genomic study, Iyer et al. (2021) showed that phage reliance on self-encoded tRNAs usually differs between phages, and their number can range between 4 and 22 per genome.

The divergence between BACPHLIP and PhageAI on the DC4 lifestyle raised the question of whether DC4 shows a prolonged propagation or pseudolysogeny/carrier state event as observed for *Pseudomonas aeruginosa* PA5oct jumbo phage (Olszak et al. 2019). Another interesting point that must be considered and could explain the discrepancy between both approaches is that these tools employ machine learning algorithms trained on a specific dataset of virulent and temperate phage genomes. Therefore, the inclusion of genomes from jumbo phages must be contemplated and the higher number of uncharacterized proteins carefully considered. The high identity (96.4%) between the DC4 *gyrA* intein and those identified in *gyrA* phages PALS2, Madawaska, and MarsHill suggest that both phages have acquired it by common ancestor as hypothesized for the phages Madawaska and MarsHill (Korn et al. 2021).

One of the interesting aspects of DC4 biology is the annotation of at least nine multi-subunit DNA-dependent RNA polymerases (RNAPs) and a Sigma70 factor, which suggests that DC4 does not rely exclusively on the host transcription machinery to express its genes. Although, the presence of multiple RNAPs is considered a common feature across the genomes of jumbo phages, and a higher diversity of virion, nonvirion RNAPs (nvRNAPs), and transcriptional factors have been reported across different jumbo viruses (Iyer et al. 2021). The presence of more than one paralogous for RNAP might reflect the use of different subunits according to the lifecycle and type of genes (early, middle, or late) that have been expressed in a certain moment (Miller et al. 2003). Interestingly, it is assumed that the presence of these multi-subunits might broaden the jumbo phages host spectrum due to their overall lower dependence on the host machinery in terms of metabolism (Yuan and Gao 2017).

Another remarkable feature encoded in the DC4 genome that can broaden the viral spectrum of hosts is a predicted RNA-dependent DNA polymerase (reverse transcriptase). This phenomenon was observed for phages that infect the genus *Bordetella*, where the selective mutagenesis of the phage tail fiber (VR1 region of the *mtd* gene) was dependent of a retron-like reverse transcriptase to extend the phage host range (Liu et al. 2004). As argued by Korn et al. (2021), its role and association with host switching in jumbo phages infecting *S*. *aureus* have not been confirmed and need further investigation mainly due to the absence of characteristic regions that would include it in the diversity-generating retroelements (DGRs) group. Interestingly, based upon nucleoside analysis conducted by Uchiyama et al. (2014) which demonstrated the presence of deoxyuridine rather than thymidine in the nucleic acid of *Staphylococcus* phage S6 and its sequence, similarity to DC4 indicates that the latter phage possesses a similar modification. Korn et al. (2021) commented that the genomes of *S*. *aureus* phages MarsHill, Madawaska, and Machias have “presumably hypermodified DNA which inhibits sequencing by several different common platforms,” though the hypermodification is not supported by the above mentioned nucleoside analysis. Analysis of the staphylococcal jumbo phage proteomes reveals that they all encode a reverse transcriptase (RNA-dependent DNA polymerase).

The in silico prediction of several bacterial hosts (obtained using HostPhinder) is a quite uncommon feature for a phage. According to Villarroel et al. (2016), this tool assumes that bacteriophages with similar genomic features are prone to share bacterial hosts. Since jumbo phages generally lack the typical modular organization found in viruses with smaller-genome sizes, a high number of uncharacterized and presumed to have uracil-substituted DNA which interferes with DNA sequencing genes (Uchiyama et al. 2014; Naknaen et al. 2021), the use of in silico tools to predict phage hosts must be considered with caution. According to the host range analysis, DC4 showed antimicrobial activity mainly against *S*. *aureus* isolated from the veterinary ecosystem, which is indeed where this virus was first isolated (da Silva Duarte et al. 2020) and may reflect its predilection to *S*. *aureus* infecting animal husbandry. It is important to stress out that due to the lack of *Bacillus* strains from veterinary sources, the capability of DC4 to infect *Bacillus* species remains to be investigated.

Besides phage DC4 presents homology both with *Bacillus* and *Staphylococcus* infecting phages, it is possible to observe that jumbo phages infecting *Staphylococcus* are closely related to jumbo viruses infecting *Bacillus*. This suggests that a set of core genes may be shared between viruses infecting both bacterial genera. Additionally, according to previous studies (Iyer et al. 2021; Weinheimer and Aylward 2022), genes related to replication machinery and infection apparatus are specifically shared between jumbo phages and small genome phages, which supports the creation of a distinct clade for jumbo phages within the class *Caudovirecetes* (tailed viruses of bacteria and archaea).

Based on the phylogenomic analysis, we further estimated the whole-genome similarity among the DC4 and other *Staphylococcus* phages (NCBI: taxid10239), by calculating the mean nucleotide identity of orthologous gene pairs shared between two viral genomes. At least four relevant clusters were grouped based on high ANI scores. Phages from different clusters exhibited extremely low or no similarity among them, which indicates a divergent origin (Yuan and Gao 2017). According to the International Committee on Taxonomy of Viruses (ICTV) (Adriaenssens and Brister, 2017), an average nucleotide identity (ANI) higher than 95% is used to classify bacteriophages at the species level. Because of this, and based on VIRIDIC and VIPtree results, DC4 phage was considered one of four new species in a new genus and shows high similarity with the phage PALS2. Notwithstanding that both viruses share high homology, it is noteworthy to mention some differences between them. The virus PALS2 was isolated from bird feces, whereas DC4 was obtained from dairy cattle farm waste (da Silva Duarte et al. 2020). Furthermore, DC4 encodes a Phe-tRNA and has an apparent narrower host spectrum among different isolates of *S*. *aureus*, while PALS2 encodes an Asn-tRNA and can infect different *Staphylococcus* species (Lee et al. 2021). As discussed by Delesalle et al. (2016), the presence of tRNA genes in bacteriophages has been justified based on codon or amino acid usage. One of the hypotheses suggests that phages with codon usages like their hosts would not retain such tRNA genes, which is not observed for the virus DC4.

The comparative analysis of orthologous genes among the six *Staphylococcus* jumbo phages revealed 303 clusters and 170 single-copy gene clusters. Regarding lytic enzymes, nine proteins on phage DC4 genomes could be aligned and present homology with other four *S*. *aureus* jumbo phages. Three of these proteins were classified as cell wall hydrolases, two have PGBD domains, one encodes a putative transglycosylase IsaA, and the last tree possessed tail-associated enzymes. Different domains were identified among the *S*. *aureus* jumbo phages lytic enzymes. Deacetylases and pectate lyase are classified as phage-encoded polysaccharide depolymerases (PSDs) that allow phages to degrade and overcome bacterial barriers such as capsular polysaccharides, exopolysaccharides, and lipopolysaccharides (Danis-Wlodarczyk et al. 2021). Pectate/pectin lyases are characterized by cleavage of the α-1,4 bonds of polygalacturonic acid and have been identified in tail proteins of bacteriophages infecting different bacterial genera such as *Salmonella*, *Acinetobacter*, and *Campylobacter* and might function in the structure of receptor-binding proteins (RBPs) (Latka et al. 2017; Oliveira et al. 2017; Thanki et al. 2019; Sørensen et al. 2021). The presence of PSD in jumbo phages is a quite common feature reported during functional annotation of viral genes. Depolymerase activity is typically verified by the presence of phage plaque-surrounding halo zones due to its production and diffusion in a soluble form. It is noteworthy mentioning that after plating and incubation for 24 h, the presence of halo zones surrounding phage plaques have been recorded for DC4, and its diameter size changed according to the host used for propagation (Fig. S5).

Bacterial peptidoglycan cleavage by hydrolases (e.g., glycosidase, amidase, and endopeptidase) is essential along the bacteriophage lifecycle and relies on the activity of specific enzymes classified as virion-associated peptidoglycan hydrolases (VAPGHs) or endolysins (Danis-Wlodarczyk et al. 2021). VAPGHs are structural components of the virion particle involved in the initial steps of phage infection and are characterized by a modular structure composed of one or two N-terminal catalytic domains (EAD, enzymatically active domain) but commonly lacking one C-terminal cell wall-binding domain (CBD); on the contrary, most staphylococcal phage endolysins display both EAD and CBD domains (Gutiérrez et al. 2018). Undoubtedly, the use of phage lytic enzymes has been one of the most studied classes of new antimicrobials against *S*. *aureus* over the last few years, mainly aimed at the control of methicillin-resistant *S*. *aureus* (MRSA) and vancomycin-resistant *S*. *aureus* (VRSA) (Duarte et al. 2021; Gutiérrez et al. 2021).

For the control of bovine mastitis caused by *S. aureus*, Donovan et al. (2006) demonstrated that the endolysin phi11 is active at the physiological pH and Ca^2+^ concentration of milk and is efficient in controlling bacterial growth in turbidity assays. When evaluated in animal models of mastitis, the phage lytic protein LysRODI outperformed CHAPSH3b displaying a pronounced activity to prevent mammary infections by *S*. *aureus* and *Staphylococcus epidermidis*. For the enzymes λSA2-E-Lyso-SH3b/λSA2-E-LysK-SH3b, a pronounced effect in terms of reducing bacterial load was also observed (Schmelcher et al. 2012). Although little information is available regarding field trials, Fan et al. (2016) report that Trx-SA1 could effectively control mild clinical mastitis caused by *S*. *aureus* in dairy cows by reducing somatic cell and pathogen levels after treatment. Overall, the identification and characterization of catalytic domains, mainly from newly isolated jumbo phages, can help in the design of new chimeric enzymes and their evaluation against *S*. *aureus* and biofilms formed by this species.

Phage structural proteome analysis has been used to better comprehend and annotate the set of proteins found on phage particles after a complete life cycle (Lavigne et al. 2009). The DC4 genome possesses 20 putative genes related to virion proteins; purified phage particles were analyzed using LCMS to verify the mature phage virion of DC4 and identified 26 peptides, including a vRNAP. The presence of vRNAP is a common feature of jumbo phages and is associated with the transcription of early genes (Ceyssens et al. 2014; Sokolova et al. 2017); however, the presence of the other three co-packaged proteins must be further confirmed since the presence of viral chaperons following viral purification using anion exchange chromatography has been reported (da Silva Duarte et al. 2018a). UvsX-like proteins are involved in homologous recombination and error-free repair of DNA double-strand breaks (Maher and Morrical 2013), whereas MoxR-like ATPase and GroEL act as chaperone-like proteins helping the maturation of metabolic protein complexes and the assembly of phage capsid proteins, respectively (Ang et al. 2000; Snider and Houry 2006).

In conclusion, bacteriophages are the most abundant organisms on Earth which makes them an incredible source of biodiversity with still many uncovered biological features to be explored. Thanks to the next-generation tools and bioinformatic pipelines, there has been a recent increase, although with still relatively very few specimens, in the number of phages classified as *S*. *aureus* jumbo phages. The virus vB_SauM-UFV_DC4 was isolated from the dairy environment in Vicosa, Minas Gerais, Brazil. This virus has a small burst size of progeny of 3 PFUs per infected cell, shows a great stability in basic pHs, and shares a high identity with the virus PALS2 (93.5%). This study reports that the viruses DC4, PALS2, MarsHill, and Madawaska can be considered viral species belonging to a new genus within the class *Caudoviricetes*. DC4 carries a set of virion and non-virion RNA polymerase subunits, an intein disrupted gene (*gyrA*), an endonuclease (VSR and LAGLIDADG), and an RNA-directed DNA polymerase. Lastly, DC4 and closely related jumbo phages showed a similar set of lytic proteins (tailed-associated or not), except for the presence of a deacetylase domain present in the tail of the phage Madawaska. The identification and characterization of catalytic domains from newly isolated jumbo phages can help in the design of new chimeric enzymes against *S*. *aureus* and biofilms formed by this species.

### Supplementary Information

Below is the link to the electronic supplementary material.

Supplementary file1 (PDF 1695 KB)

## Data Availability

The authors declare that sequence data that support the findings of this study have been deposited in GenBank with the accession number MZ779063. All other data supporting the findings of this study are available within the article and its supplementary information files.
