# Supplementary material for: Genomic and proteomic characterization of vB_SauM-UFV_DC4, a novel Staphylococcus jumbo phage
Source: Appl Microbiol Biotechnol. 2023 Sep 23;107(23):7231–50. doi: 10.1007/s00253-023-12743-6 (PMC10638138; doi:10.1007/s00253-023-12743-6)

Genomic and proteomic characterization of vB_SauM-UFV_DC4, a novel *Staphylococcus* jumbo phage

Jéssica Duarte da Silva[1], Luís D. R. Melo[2,3], Sílvio B. Santos[2,3], Andrew M. Kropinski[4], Mariana Fonseca Xisto[5], Roberto Sousa Dias[5], Isabela da Silva Paes[5], Marcella Silva Vieira[5], José Júnior Ferreira Soares[5], Davide Porcellato[6], Vinícius da Silva Duarte[1,6], Sérgio Oliveira de Paula[5]

[1] Department of Microbiology, Federal University of Viçosa, Av. Peter Henry Rolfs, s/n, Campus Universitário, 36570-900, Viçosa, Minas Gerais, Brazil

[2] Centre of Biological Engineering – CEB, University of Minho, 4710-057, Braga, Portugal

[3] LABBELS – Associate Laboratory, Braga, Portugal

[4] Department of Pathobiology, University of Guelph, Guelph, Ontario, N1G 2W1, Canada

[5] Department of General Biology, Federal University of Viçosa, Av. Peter Henry Rolfs, s/n, Campus Universitário, 36570-900, Viçosa, Minas Gerais, Brazil

[6] Faculty of Chemistry, Biotechnology and Food Science, The Norwegian University of Life Sciences, P.O. Box 5003, 1432, Ås, Norway

*Corresponding author: vinicius.da.silva.duarte@nmbu.no

Table S1 – *S. aureus* isolates and their corresponding source. Positive and negative stand, respectively, for the identification or not of lysis plaques following spot test assay.

**Source of isolation**	**Isolate**	**vB_SauM_UFV_DC4**
Human hospital	*S. aureus* 10	-
	S. aureus 67	-
	S. aureus 112	-
	S. aureus 261	+
Veterinary hospital	*S. aureus* 552	-
	S. aureus 574 HV	+
	S. aureus 607 HV	-
	S. aureus 724.2	-
	S. aureus 3059	+
	S. aureus 3907	+
	S. aureus 1334	+
	S. aureus ATCC 33591	+
	S. aureus 222	+
	S. aureus O46	+
	S. aureus 3.2	+
	S. aureus 32/2	+
	S. aureus 4182	+
	S. aureus 4081	+
Meat products	*S. aureus* 3	-
	S. aureus 145.2	-
	S. aureus 148.1	-
	S. aureus 177.2	-
Environment	*S. aureus* 25.1	-
	S. aureus 84.1	-
	S. aureus 178.2	-
	S. aureus 181.1	-

Table S2 – Detailed information of the 83 whole-genome sequences (74 *Staphylococcus* phages and 9 *Bacillus* phages) downloaded from the National Center for Biotechnology Information (NCBI:taxid10239) using inphared (INfrastructure for a PHAge REference Database, release 3Oct2021).

Accession	Description	Genome Length (bp)	Jumbo phage	Mol GC (%)	Number CDS	Coding Capacity (%)	tRNAs	Host	Genus	Family
EF583821	Bacillus phage 0305phi8-36	218948	TRUE	41,8	245	94,8	0	Bacillus	Unclassified	Myoviridae
JN638751	Bacillus virus G	497513	TRUE	29,9	669	87,7	17	Bacillus	Donellivirus	-
KT624200	Bacillus phage SP-15	221908	TRUE	38,6	310	90,8	0	Bacillus	Thornevirus	-
KT895374	Bacillus phage vB_BpuM-BpSp	255569	TRUE	25,9	314	91,6	0	Bacillus	Takahashivirus	-
KU878088	Bacillus phage AR9	251042	TRUE	27,8	296	89,6	1	Bacillus	Takahashivirus	-
LC597490	Bacillus phage vB_BceM_WH1	229829	TRUE	37,2	299	93,2	0	Bacillus	Unclassified	Myoviridae
MF360957	Bacillus virus PBS1	252197	TRUE	27,7	299	89,8	1	Bacillus	Takahashivirus	-
MN091626	Staphylococcus phage PALS_2	268748	TRUE	25,1	274	91,8	1	Staphylococcus	Unclassified	Myoviridae
MW218148	Staphylococcus phage vB_StaM_SA1	260727	TRUE	26,8	256	92,6	0	Staphylococcus	Unclassified	Myoviridae
MW248466	Staphylococcus phage MarsHill	266637	TRUE	25,2	267	91,1	0	Staphylococcus	Unclassified	Myoviridae
MW349128	Staphylococcus phage Machias	274478	TRUE	24,8	283	89,9	1	Staphylococcus	Unclassified	Myoviridae
MW349129	Staphylococcus phage Madawaska	265446	TRUE	25,1	267	91,5	1	Staphylococcus	Unclassified	Myoviridae

MW749003	Bacillus phage vB_BspM_Internexus	252262	TRUE	27,7	291	88,4	1	Bacillus	Takahashivirus	-
MW749006	Bacillus phage vB_BspM_AgentSmith	200233	TRUE	37,0	210	92,8	2	Bacillus	Unclassified	Myoviridae
MZ779063	Staphylococcus phage vB_SauM-UFV_DC4	263185	TRUE	25,0	261	91,9	1	Staphylococcus	Unclassified	Myoviridae
AB626962	Staphylococcus phage S24-1	18168	FALSE	28,9	21	94,2	0	Staphylococcus	Rosenblumvirus	Rountreeviridae
AB626963	Staphylococcus phage S13'	18186	FALSE	29,2	21	94,0	0	Staphylococcus	Rosenblumvirus	Rountreeviridae
AF513032	Staphylococcus virus 44AHJD	16784	FALSE	29,6	20	93,4	0	Staphylococcus	Rosenblumvirus	Rountreeviridae
AF513033	Staphylococcus phage P68	18227	FALSE	29,3	20	93,5	0	Staphylococcus	Rosenblumvirus	Rountreeviridae
AY954949	Staphylococcus phage 66	18199	FALSE	29,3	21	92,6	0	Staphylococcus	Rosenblumvirus	Rountreeviridae
CP018841	Staphylococcus phage HOB 14.1.R1	18659	FALSE	32,9	37	89,4	0	Staphylococcus	Unclassified	Siphoviridae
DQ834250	Staphylococcus virus PH15	44041	FALSE	34,9	72	93,2	0	Staphylococcus	Rockefellervirus	Siphoviridae
EF462198	Staphylococcus phage tp310-2	47785	FALSE	33,8	69	93,2	0	Staphylococcus	Triavirus	-
EU136189	Staphylococcus phage SAP-2	17938	FALSE	28,9	20	93,3	0	Staphylococcus	Rosenblumvirus	Rountreeviridae
HF937074	Staphylococcus phage PSa3	17602	FALSE	29,6	19	94,0	0	Staphylococcus	Rosenblumvirus	Rountreeviridae
JN192400	Staphylococcus virus IPLA5	43581	FALSE	34,7	75	95,0	0	Staphylococcus	Rockefellervirus	Siphoviridae

JN192401	Staphylococcus virus IPLA7	42123	FALS E	34,8	65	95,4	0	Staphylococcus	Rockefellervirus	Siphovirida e
JQ957932	Staphylococcus phage StauST398-2	45572	FALS E	33,3	61	92,6	0	Staphylococcus	Triavirus	Siphovirida e
KF589919	Staphylococcus phage phiRS7	43394	FALS E	34,3	63	92,8	0	Staphylococcus	Unclassified	Siphovirida e
KF598856	Staphylococcus phage YMC/09/04/R1988	44459	FALS E	33,4	61	92,1	0	Staphylococcus	Triavirus	Siphovirida e
KF929199	Staphylococcus phage vB_SepS_SEP9	92417	FALS E	29,6	128	87,1	1	Staphylococcus	Sextaecvirus	Siphovirida e
KJ210330	Staphylococcus phage GRCS	17869	FALS E	28,9	20	92,1	0	Staphylococcus	Rosenblumvirus	Rountreevir idae
KJ804259	Staphylococcus phage 6ec	93794	FALS E	29,3	139	88,6	1	Staphylococcus	Sextaecvirus	Siphovirida e
KM36610 0	Staphylococcus phage BP39	17641	FALS E	29,0	21	94,2	0	Staphylococcus	Rosenblumvirus	Rountreevir idae
KP735928	Staphylococcus phage IME-SA4	41843	FALS E	34,0	65	96,2	0	Staphylococcus	Unclassified	Siphovirida e
KT429160	Staphylococcus phage SPbeta-like	127726	FALS E	30,5	156	88,4	0	Staphylococcus	Unclassified	Siphovirida e
KU99291 1	Staphylococcus phage SLPW	17861	FALS E	29,4	22	91,5	0	Staphylococcus	Rosenblumvirus	Rountreevir idae
KX15676 2	Staphylococcus phage vB_SauS_IMEP5	44677	FALS E	34,3	68	92,9	0	Staphylococcus	Unclassified	Siphovirida e
KY00008 4	Staphylococcus phage SCH1	18023	FALS E	29,3	20	94,5	0	Staphylococcus	Rosenblumvirus	Rountreevir idae
KY00008 5	Staphylococcus phage SCH111	18018	FALS E	29,3	21	94,2	0	Staphylococcus	Rosenblumvirus	Rountreevir idae
KY44206 3	Staphylococcus phage Andhra	18546	FALS E	29,8	20	94,2	0	Staphylococcus	Andhravirus	Rountreevir idae

KY471386	Staphylococcus phage St 134	18275	FALSE	30,1	20	95,4	0	Staphylococcus	Andhravirus	Rountreeviridae
KY653120	Staphylococcus phage IME1348_01	42371	FALSE	34,7	65	96,3	0	Staphylococcus	Rockefellervirus	Siphoviridae
KY653124	Staphylococcus phage IME1367_01	43135	FALSE	33,7	69	91,0	0	Staphylococcus	Unclassified	Siphoviridae
KY653126	Staphylococcus phage IME1354_01	42706	FALSE	34,2	64	96,0	0	Staphylococcus	Unclassified	Siphoviridae
MG543995	Staphylococcus phage UPMK_1	152788	FALSE	31,9	160	71,8	0	Staphylococcus	Unclassified	Siphoviridae
MG766218	Staphylococcus phage vB_SauP_phiAGO1.3	17603	FALSE	29,0	19	92,3	0	Staphylococcus	Rosenblumvirus	Rountreeviridae
MG766219	Staphylococcus phage vB_SauP_phiAGO1.9	17637	FALSE	29,0	19	92,3	0	Staphylococcus	Rosenblumvirus	Rountreeviridae
MK075005	Staphylococcus phage phiSP119-2	40011	FALSE	34,1	60	93,6	0	Staphylococcus	Unclassified	Siphoviridae
MK075006	Staphylococcus phage phiSP119-3	44311	FALSE	35,6	65	91,2	1	Staphylococcus	Unclassified	Siphoviridae
MT423823	Staphylococcus virus pSp_SNUABM-J	40224	FALSE	35,7	71	94,0	0	Staphylococcus	Unclassified	Siphoviridae
MT596497	Staphylococcus phage 456	43393	FALSE	34,7	73	91,6	0	Staphylococcus	Rockefellervirus	-
MT596498	Staphylococcus phage vB_SepS_BE01	42718	FALSE	34,8	70	94,3	0	Staphylococcus	Rockefellervirus	-
MT596499	Staphylococcus phage vB_SepS_BE02	95233	FALSE	29,4	140	87,9	1	Staphylococcus	Sextaecvirus	-
MT724048	Staphylococcus phage SAP3	41949	FALSE	35,4	64	93,9	0	Staphylococcus	Rockefellervirus	-
MT880872	Staphylococcus phage PhiSepi-HH3	147057	FALSE	30,8	170	88,3	0	Staphylococcus	Unclassified	Siphoviridae

MW221967	Staphylococcus phage PMBT9	41623	FALSE	36,2	65	94,8	0	Staphylococcus	Rockefellervirus	-
MZ488273	Staphylococcus phage vB_SarS_BM31	42271	FALSE	34,6	67	94,6	0	Staphylococcus	Unclassified	Siphoviridae
NC_002661	Staphylococcus virus phiSLT	42942	FALSE	33,3	68	93,0	0	Staphylococcus	Triavirus	-
NC_004616	Staphylococcus virus phi12	44970	FALSE	33,3	63	93,1	0	Staphylococcus	Triavirus	-
NC_004740	Staphylococcus phage phiN315	44082	FALSE	32,8	63	87,6	0	Staphylococcus	Unclassified	Siphoviridae
NC_007051	Staphylococcus phage 2638A	41318	FALSE	36,9	55	92,6	0	Staphylococcus	Fibralongavirus	Siphoviridae
NC_007052	Staphylococcus virus 42e	45861	FALSE	33,7	66	92,3	0	Staphylococcus	Triavirus	-
NC_007053	Staphylococcus virus 3a	43095	FALSE	33,5	62	94,1	0	Staphylococcus	Triavirus	-
NC_007054	Staphylococcus virus 47	44777	FALSE	33,5	61	92,8	0	Staphylococcus	Triavirus	-
NC_011344	Staphylococcus phage phi2958PVL	47342	FALSE	33,0	64	93,3	0	Staphylococcus	Triavirus	-
NC_011612	Staphylococcus virus IPLA35	45344	FALSE	33,2	63	93,1	0	Staphylococcus	Triavirus	-
NC_028821	Staphylococcus phage StB20-like	40670	FALSE	33,3	59	91,4	0	Staphylococcus	Unclassified	Siphoviridae
NC_028862	Staphylococcus phage vB_SauS_phi2	44222	FALSE	33,7	60	93,8	0	Staphylococcus	Triavirus	Siphoviridae
NC_031241	Staphylococcus phage CNPx	43293	FALSE	34,7	69	95,3	0	Staphylococcus	Rockefellervirus	Siphoviridae
NC_048192	Staphylococcus phage vB_SpsS_QT1	43029	FALSE	36,9	55	93,2	0	Staphylococcus	Fibralongavirus	Siphoviridae

NC_055038	Staphylococcus phage SH-St 15644	45111	FALSE	33,3	63	93,2	0	Staphylococcus	Triavirus	-
NC_055039	Staphylococcus phage vB_SauS_fPfSau02	45108	FALSE	33,7	69	93,3	0	Staphylococcus	Triavirus	-
NC_055040	Staphylococcus phage vB_SauS_JS02	46435	FALSE	33,1	67	91,5	0	Staphylococcus	Triavirus	-
NC_055041	Staphylococcus phage LH1	46048	FALSE	33,2	61	93,1	0	Staphylococcus	Triavirus	-
NC_055042	Staphylococcus phage P240	45985	FALSE	33,1	66	92,9	0	Staphylococcus	Triavirus	-
NC_055043	Staphylococcus phage SA137ruMSSAST121PVL	45999	FALSE	33,3	62	93,2	0	Staphylococcus	Triavirus	-
NC_055044	Staphylococcus phage SAP11	45346	FALSE	33,4	64	89,7	0	Staphylococcus	Triavirus	-
NC_055045	Staphylococcus phage phiSa2wa_st1	45585	FALSE	33,3	61	94,2	0	Staphylococcus	Triavirus	-
NC_055046	Staphylococcus phage phiSa2wa_st5	44823	FALSE	33,4	72	94,9	0	Staphylococcus	Triavirus	-
NC_055047	Staphylococcus phage phiSa2wa_st30	45780	FALSE	33,4	60	93,5	0	Staphylococcus	Triavirus	-
NC_055048	Staphylococcus phage phiSa2wa_st78	45878	FALSE	33,2	65	93,8	0	Staphylococcus	Triavirus	-
NC_055049	Staphylococcus phage phiSa2wa_st121mssa	45621	FALSE	33,1	61	94,2	0	Staphylococcus	Triavirus	-

Table S3 – Correlation analysis between selected variables obtained from inphared (INfrastructure for a PHAge REference Database, release 3Oct2021) for the 83 whole-genome sequences (74 *Staphylococcus* phages and 9 *Bacillus* phages, Supplementary Table 2).

Parameter1	Parameter2	r	95% CI	t(72)	p
Genome size (kb)	GC	-0.59	-0.72, -0.41	-6.15	< .001
Genome size (kb)	CDS	0.99	0.98, 0.99	50.89	< .001
Genome size (kb)	Genes (+ strand)	0.07	-0.16, 0.30	0.61	> .999
Genome size (kb)	Genes (- strand)	-0.07	-0.30, 0.16	-0.61	> .999
Genome size (kb)	Coding capacity	-0.36	-0.54, -0.14	-3.23	0.020
Genome size (kb)	tRNAs	0.59	0.42, 0.72	6.28	< .001
GC	CDS	-0.50	-0.65, -0.30	-4.85	< .001
GC	Genes (+ strand)	0.45	0.25, 0.61	4.26	< .001
GC	Genes (- strand)	-0.45	-0.61, -0.25	-4.26	< .001
GC	Coding capacity	0.21	-0.02, 0.41	1.78	0.552
GC	tRNAs	-0.47	-0.63, -0.27	-4.54	< .001
CDS	Genes (+ strand)	0.11	-0.12, 0.33	0.92	> .999
CDS	Genes (- strand)	-0.11	-0.33, 0.12	-0.92	> .999
CDS	Coding capacity	-0.37	-0.55, -0.16	-3.40	0.013
CDS	tRNAs	0.62	0.46, 0.75	6.79	< .001
Genes (+ strand)	Negative	-1.00	-1.00, -1.00	-Inf	< .001
Genes (+ strand)	Coding capacity	0.30	0.08, 0.49	2.66	0.096
Genes (+ strand)	tRNAs	-0.09	-0.31, 0.14	-0.78	> .999
Genes (- strand)	Coding capacity	-0.30	-0.49, -0.08	-2.66	0.096
Genes (- strand)	tRNAs	0.09	-0.14, 0.31	0.78	> .999
Coding capacity	tRNAs	0.28	-0.48, -0.05	-2.45	0.133

Figure S1 - BLAST Ring Image Generator (BRIG) diagram showing the complete genome of five jumbo phages (colored concentric rings) infecting *S. aureus*. The phage DC4 served as a reference. The innermost circles represent the GC content (black) and GC skew (purple/green) of DC4 phage genome.

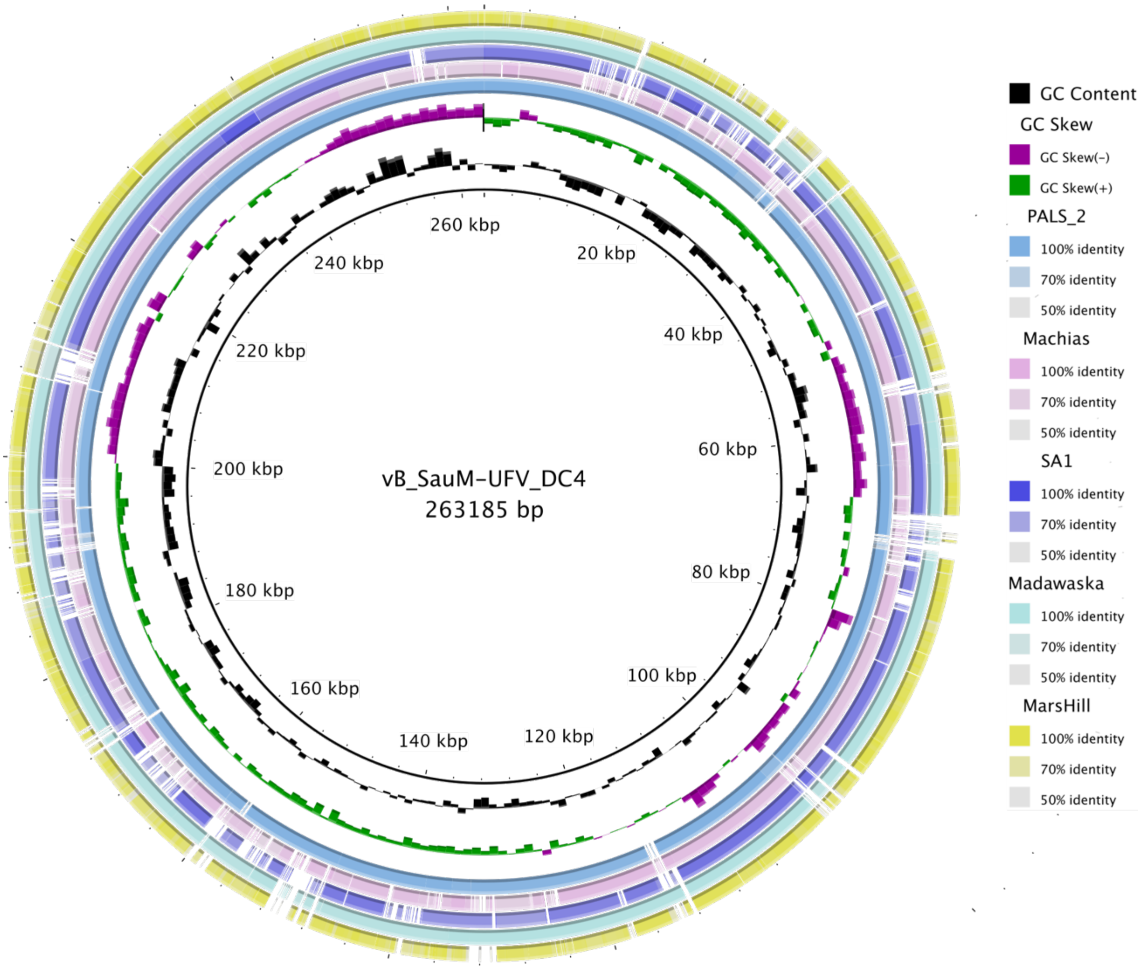

Figure S2 – Genome alignment with progressive Mauve showing two Co-linear Blocks (LCB) between the viruses PALS2 and DC4. Mean pairwise identity over all pairs in the column: Green: 100% identity; Greeny-brown: 30% - 100% identity; Red: below 30% identity.

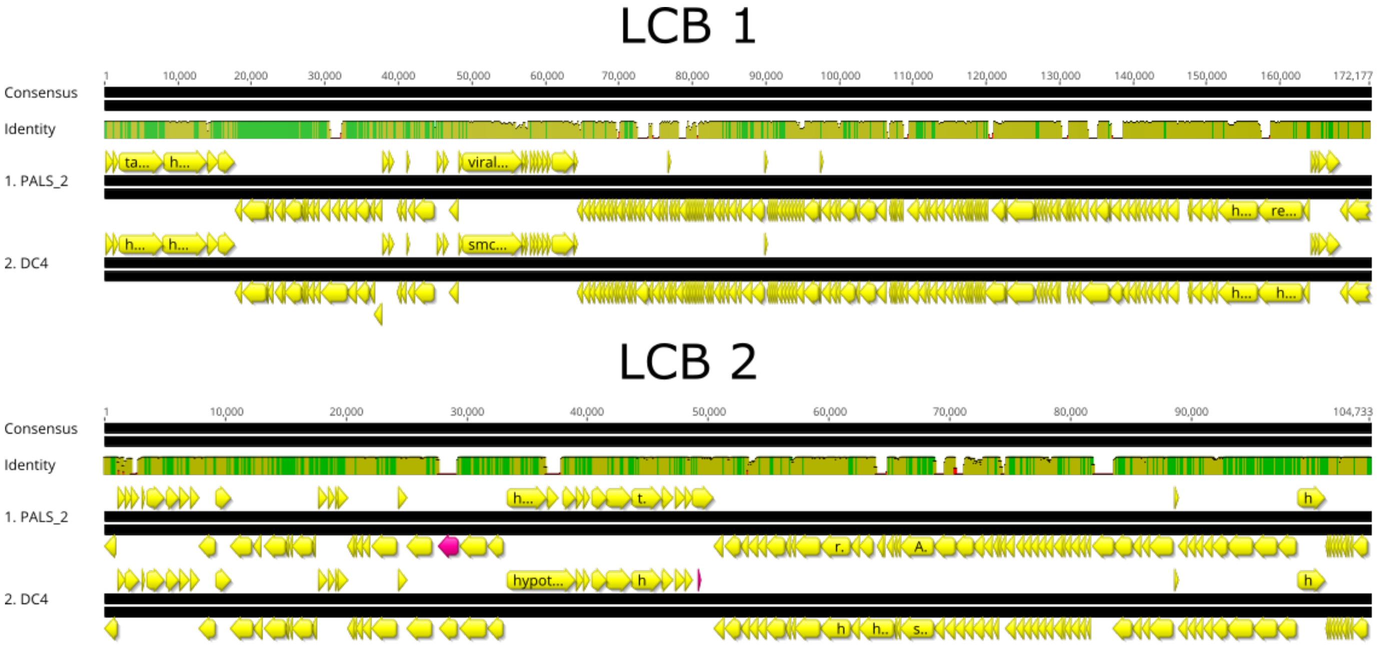

Figure S3 – Frequency of codon usage based on the genomes of the phage DC4 and its hosts *S. aureus* 2030RH1 and 3059.

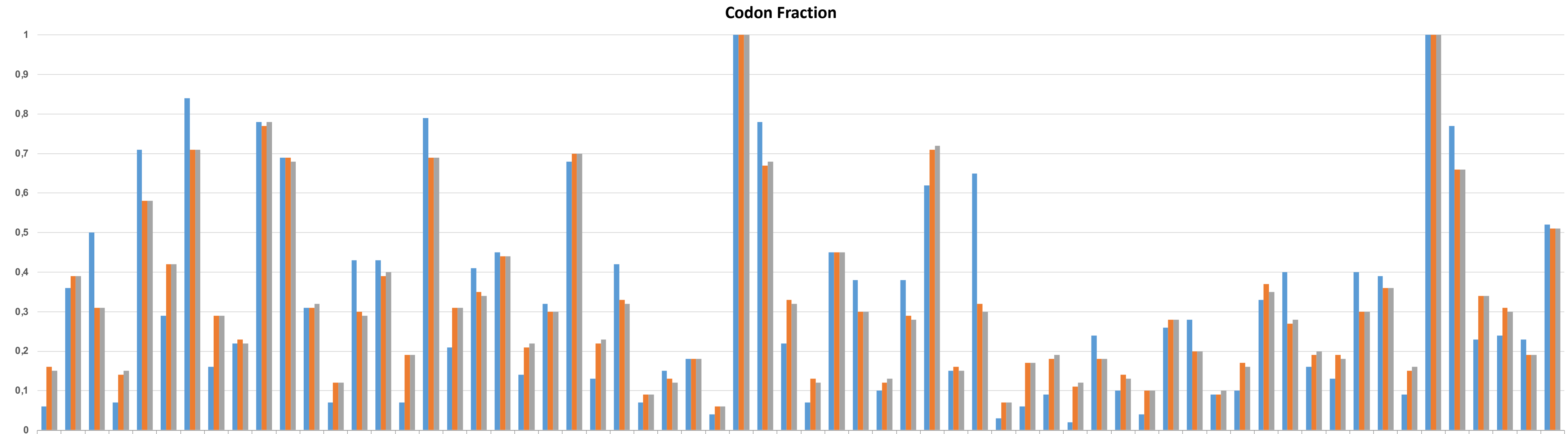

	GCG	GCA	GCT	GCC	TGT	TGC	GAT	GAC	GAG	GAA	TTT	TTC	GGG	GGA	GGT	GGC
DC4	0,06	0,36	0,5	0,07	0,71	0,29	0,84	0,16	0,22	0,78	0,69	0,31	0,07	0,43	0,43	0,07
2030	0,16	0,39	0,31	0,14	0,58	0,42	0,71	0,29	0,23	0,77	0,69	0,31	0,12	0,3	0,39	0,19
3059	0,15	0,39	0,31	0,15	0,58	0,42	0,71	0,29	0,22	0,78	0,68	0,32	0,12	0,29	0,4	0,19

	CAT	CAC	ATA	ATT	ATC	AAG	AAA	TTG	TTA	CTG	CTA	CTT	CTC	ATG	AAT	AAC
DC4	0,79	0,21	0,41	0,45	0,14	0,32	0,68	0,13	0,42	0,07	0,15	0,18	0,04	1	0,78	0,22
2030	0,69	0,31	0,35	0,44	0,21	0,3	0,7	0,22	0,33	0,09	0,13	0,18	0,06	1	0,67	0,33
3059	0,69	0,31	0,34	0,44	0,22	0,3	0,7	0,23	0,32	0,09	0,12	0,18	0,06	1	0,68	0,32

	CCG	CCA	CCT	CCC	CAG	CAA	AGG	AGA	CGG	CGA	CGT	CGC	AGT	AGC	TCG	TCA
DC4	0,07	0,45	0,38	0,1	0,38	0,62	0,15	0,65	0,03	0,06	0,09	0,02	0,24	0,1	0,04	0,26
2030	0,13	0,45	0,3	0,12	0,29	0,71	0,16	0,32	0,07	0,17	0,18	0,11	0,18	0,14	0,1	0,28
3059	0,12	0,45	0,3	0,13	0,28	0,72	0,15	0,3	0,07	0,17	0,19	0,12	0,18	0,13	0,1	0,28

	TCT	TCC	ACG	ACA	ACT	ACC	GTG	GTA	GTT	GTC	TGG	TAT	TAC	TGA	TAG	TAA
DC4	0,28	0,09	0,1	0,33	0,4	0,16	0,13	0,4	0,39	0,09	1	0,77	0,23	0,24	0,23	0,52
2030	0,2	0,09	0,17	0,37	0,27	0,19	0,19	0,3	0,36	0,15	1	0,66	0,34	0,31	0,19	0,51
3059	0,2	0,1	0,16	0,35	0,28	0,2	0,18	0,3	0,36	0,16	1	0,66	0,34	0,3	0,19	0,51

Figure S4 – Comparative genome analysis for *S. aureus* jumbo phages performed by the OrthoVenn2 webserver. Venn diagram showing the distribution of shared gene families (orthologous clusters) among the six *S. aureus* jumbo phages (MarsHill, Machias, PALS2, DC4, Madawaska, and SA1). The cluster number in each component is listed.

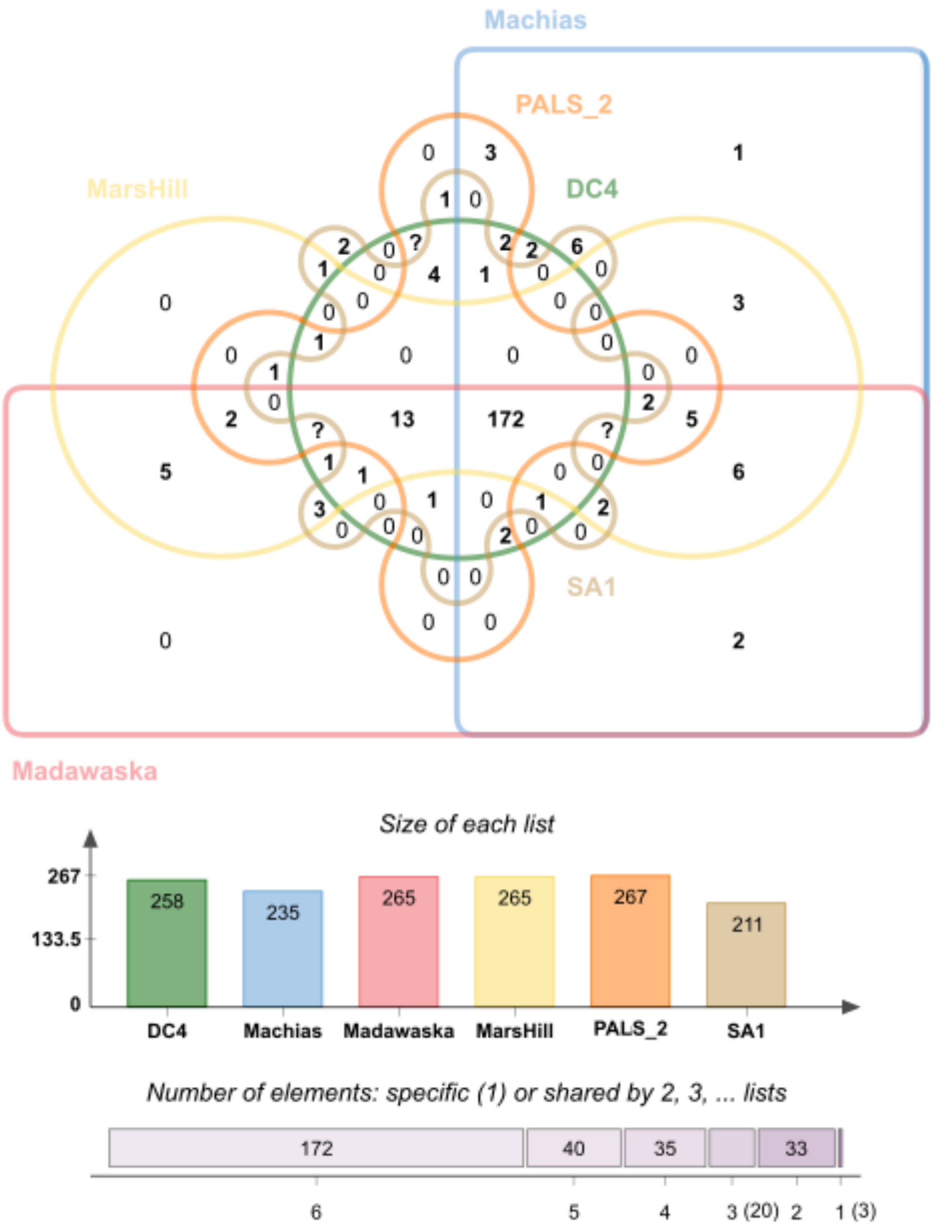

Figure S5 – DC4 plaque formed in double-layer agar displays a clear lytic zone (arrows) with a surrounding halo indicative of phage-derived enzymes able to destroy bacterial cell wall components according to the host of incubation (arrowhead – **A, B**, and **E**). A – *S. aureus* O46; B – *S. aureus* 222; *S. aureus* 574 HV; *S. aureus* 1334; *S. aureus* 32/2; *S. aureus* 3907.

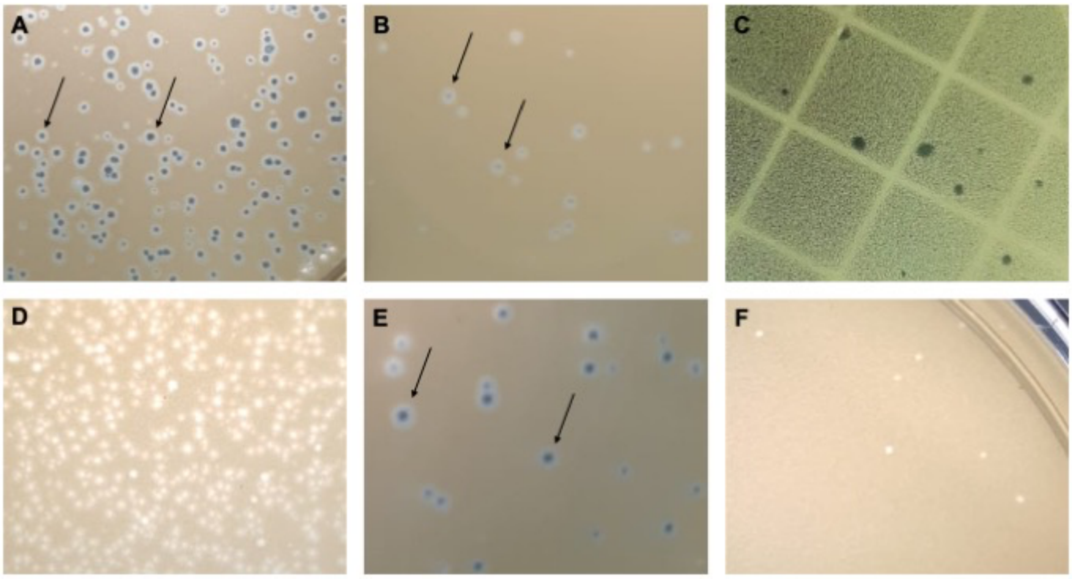

Supplement: Supplementary file 1 — Supplementary file1 (PDF 1695 KB) [file 253_2023_12743_MOESM1_ESM.pdf]
